# Scarce and directly beneficial reputations support cooperation

**DOI:** 10.1038/s41598-020-68123-x

**Published:** 2020-07-13

**Authors:** Flóra Samu, Szabolcs Számadó, Károly Takács

**Affiliations:** 1 The Institute for Analytical Sociology, Linköping University, 601 74 Norrköping, Sweden; 2 Doctoral School of Sociology, Corvinus University of Budapest, Fővám tér 8, Budapest, 1018 Hungary; 3 Centre for Social Sciences (TK CSS) ‘Lendület’ Research Center for Educational and Network Studies (CSS-RECENS), Tóth Kálmán u. 4, Budapest, 1097 Hungary; 4 Department of Sociology and Communication, Budapest University of Technology and Economics, Egry J. u. 1, Budapest, 1111 Hungary; 5 Evolutionary Systems Research Group, Centre for Ecological Research, Klebelsberg Kuno u. 3, Tihany, 8237 Hungary

**Keywords:** Social evolution, Human behaviour

## Abstract

A human solution to the problem of cooperation is the maintenance of informal reputation hierarchies. Reputational information contributes to cooperation by providing guidelines about previous group-beneficial or free-rider behaviour in social dilemma interactions. How reputation information could be credible, however, remains a puzzle. We test two potential safeguards to ensure credibility: (i) reputation is a scarce resource and (ii) it is not earned for direct benefits. We test these solutions in a laboratory experiment in which participants played two-person Prisoner’s Dilemma games without partner selection, could observe some other interactions, and could communicate reputational information about possible opponents to each other. Reputational information clearly influenced cooperation decisions. Although cooperation was not sustained at a high level in any of the conditions, the possibility of exchanging third-party information was able to temporarily increase the level of strategic cooperation when reputation was a scarce resource and reputational scores were directly translated into monetary benefits. We found that competition for monetary rewards or unrestricted non-monetary reputational rewards helped the reputation system to be informative. Finally, we found that high reputational scores are reinforced further as they are rewarded with positive messages, and positive gossip was leading to higher reputations.

## Introduction

Cooperation is integral part of our daily life^1,2^. In cooperation situations, however, there is a conflict between individual and common interests^3^. The most severe cases is the Prisoner’s Dilemma (PD) game^4^ in which following selfish interests is the dominant strategy that disallows the establishment of the collectively optimal cooperation outcome that is superior for every interaction partner compared to mutual defection. Over the past decades a wide range of proposals have been made how to resolve the problem of cooperation^5,6^. One of the informal solutions proposed by the theory of indirect reciprocity (IR) is the establishment and maintenance of reputations that provide guidelines for selecting the right partners but also for distinctive actions towards interaction partners^7–9^. Empirical studies confirmed that cooperation can be established through the use of reputations that trigger conditional cooperative behaviour^10–14^.

In previous empirical studies, where reputations were shown to provide an efficient solution for social dilemmas, individuals could observe the past behaviour of others and hence they had perfect and true information on who had been cooperating and who had not^15,16^. In large populations, however, it is not feasible to observe past decisions of potential unknown transaction partners directly and a credible summary score is not always publicly available. The mechanism that helps to access reputational information is gossip in which individuals exchange evaluative third-party information^17^. Seminal models (and reviews) of the IR paradigm operate with the assumption^7,8,18–20^ that gossip needs to be reliable to ensure that information received is attended and to ensure that reputation reflect past action^5^.

There are, however, unresolved puzzles around the reliability of gossip. On the one hand, empirical observations show that humans lie^21,22^ and that gossip could be used to undermine the target’s reputation strategically^23,24^. Incorporating the option to send strategically dishonest messages in an IR model in fact leads to the collapse of cooperation^25^. From the perspective of strategic motivations, dishonesty could be pro self^21,22,24^ or prosocial^26^. For example, people can lie to improve their own reputation, to destroy the reputation of their competitors or to serve the interest of their group^26,27^.

On the other hand, dice-roll experiments^30^ consistently show that people do not lie as much as expected based on the utility maximizing “homo economicus” paradigm; i.e. “they leave much on the table”^31^. The preference of being honest was one of the main factors behind this “truth seeking” behaviour. Similarly, research on strong reciprocity^32–34^ proved that negative emotional reactions to selfish behaviour can lead to altruistic punishment^35,36^. Correspondingly, prosocial gossip operates with similar underlying negative emotions and it can be used to punish (and deter) selfish behavior^37^. Moreover, in experiments where participants could gossip, transmitted information was very much in line with observed choices^12,38–40^. Gossip was observed even when it implied substantial costs for the sender^41^. Furthermore, studies suggest that gossip does not need to be completely accurate; even with noises, it can promote trust and cooperation^28,29^. Last but not least, spreading reputational information honestly may involve additional advantages to the sender^42,43^, such as an increased reputation for reliability. The Supplementary Table S1, in A1 summarizes the proposed explanations behind honest vs. dishonest gossip.

The processing of third-party information is an important element of a functional reputation system. The rules regulating the assignment of reputation based on information available for the individual are called social norms^44,45^. First order social norms are conditional on the previous observed action only, for instance considering cooperators good and defectors bad^7^. Higher order norms take into account also the reputation of the opponent in an observed action of another player. Cooperation is difficult to be maintained by first order social norms. Certain second and third order social norms work better as they allow justified defection, i.e., the punishment of previous defectors by defection^18–20,46^. Social norms have mainly been analysed in models assuming unbiased and public reputations and homogenous populations^18–20^, although the investigation has been extended also to hypocritical strategies and private situations^47^. In an exploration of possible social norms, eight norms (“*leading eight*”) has been found to be able to sustain cooperation^18–20^. It is still an open question which of these social norms could be observed in empirical situations.

**Figure 1:**
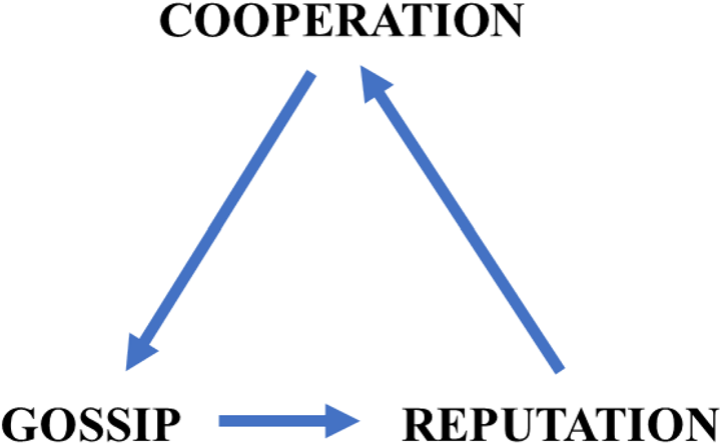
Schematic representation of the relationship between cooperation, gossip, and reputation.

In line with the literature that recognizes the importance of gossip and reputation for cooperation, we expect that where gossip is available, it will provide relevant information on partners that enable cooperation condition on the partner’s reputation. The alleged relationships between gossip, reputations, and cooperation are displayed in Fig. 1.

### H1

The possibility of gossip increases cooperation.

In line with the theoretical literature on reputational systems we assume that: (i) individuals use gossip to transmit their direct observations to others; (ii) reputational scores will be updated based on the information received. Therefore, we put the following mechanisms forward as sub-hypotheses:

### H1a

Gossip will be in line with partners’ previous decisions.

### H1b

Reputations are updated in the direction of the valence of gossip received.

Under which conditions reputations could facilitate cooperation, however, is an open question. We propose that reputations communicated via gossip could increase cooperation if reputation is a scarce resource and hence a competitive frame is created. The theory of ‘competitive altruism’^48–50^ asserts that competition is the motivation for reputation-building which incorporates in the rise of pro-social behaviour. Hence, individuals compete for being more cooperative than others in order to keep up with their reputation^51–54^. When the highest reputation cannot be gained by everyone there is more motivation for investments in acquiring good reputation, because the individual’s relative position depends on others behaviour. Therefore, we expect that individuals cooperate more in order to avoid the weakening of their relative reputation in the eye of others.

### H2a

Competition for scarce reputations increases cooperation.

On the other hand, relative position can be improved also by undermining the reputation of competitors (sharing negative gossip about them)^24^^.^^55,56^. So far, only competition for mates was tested empirically where romantic rivalry was taken for granted^57,58^, but there is no empirical evidence where rivalry is independent of gender. In a competitive environment for reputation, dishonest strategic gossip could also occur more likely, while there are no motivations for sending positive gossip dishonestly.

### H2b

Competition for scarce reputations increases negative gossiping.

As another mechanism, we propose that monetary stakes for reputation have negative effect on cooperation. Beyond partner selection, good reputation might help individuals acquiring other beneficial outcomes such as additional resources^42^, greater influence^59^, or social network benefits^60,61^. Even if tangible incentives can foster cooperation simply because they reduce the magnitude of conflict between self-interest and the common good^62^, empirical studies have shown that external incentives can reduce the motivation for reputation-building^63–66^ and as a result, the level of cooperation does not grow as much as we would observe in the absence of this ‘crowding-out’ or ‘overjustification’ effect^67,68^. Either punishment as an external incentive^69,70^ or rewards^71^ can reduce motivation to achieve high reputation. The mechanism behind the reduced motivation for reputation-building supposed to be the lack of opportunity to signalling group-based motivation or commitment^43,61,72–75^. We expect that if direct external incentives are linked to the reputational position, then the signal of long-term commitment or group-based motivation will be inseparable from the motives for direct benefits^43,61,74^. In this case reputational signals will be less efficient, which could directly be traced in the distribution of reputations and impact cooperation as a consequence.

### H3

Direct monetary stakes for reputation decrease cooperation.

**Figure 2:**
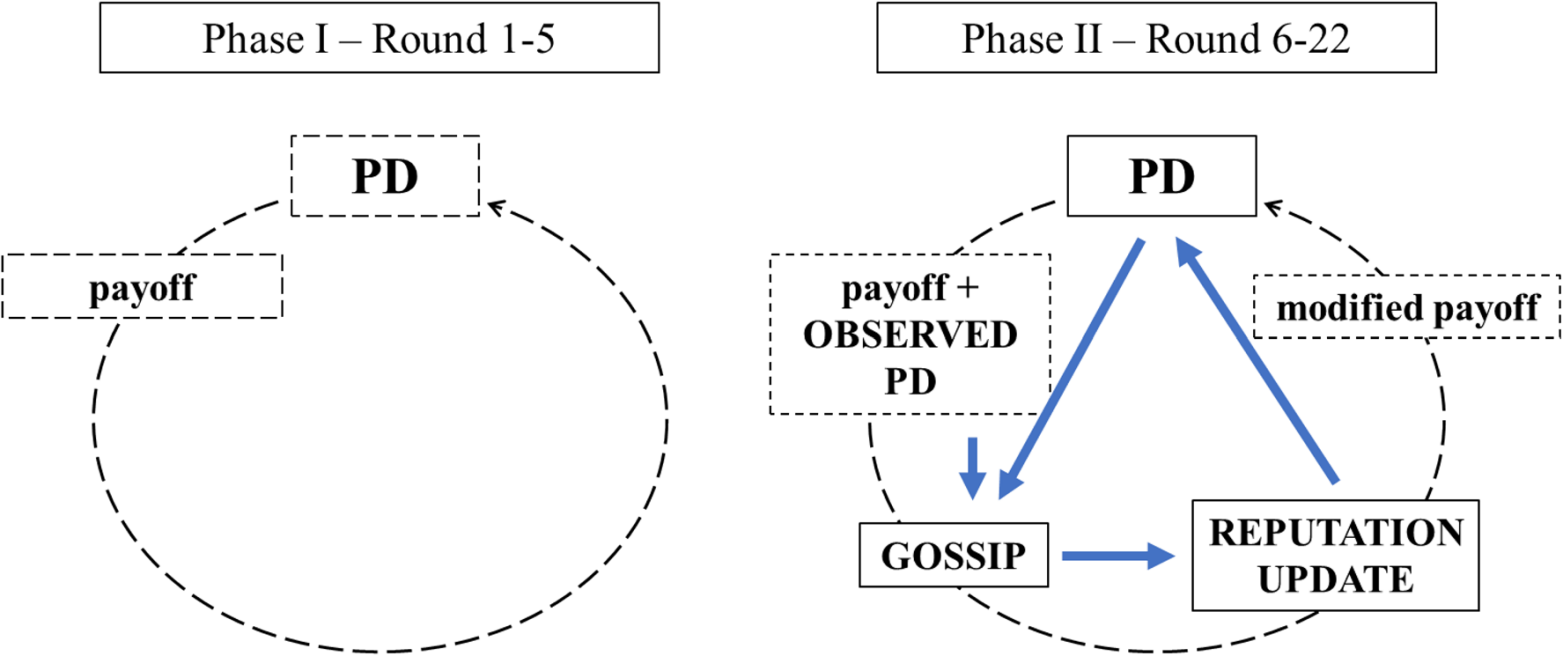
Description of one round in our experiment. Participants played two two-person Prisoner’s Dilemma games in each round in Phase I (control) and in addition, the opportunity of gossiping and scoring others’ reputation were introduced in Phase II.

We aim to show how extrinsic motivation and competition for scarce resources affect strategic reputation building and cooperation in an environment where there is a low probability of meeting with the same person again. We test whether the degree of competition influences the level of cooperation in a two-person Prisoner’s Dilemma game by manipulating the scarcity of the reputational resources (H2) and further monetary incentives for reputation-building (H3). The main part of the experiment follows a 2 × 2 between-subject design. The scarcity of reputations is manipulated by the way participants can distribute reputation scores to others (on a scale between 0 and 100). We call treatments *abundance (A* from now on) where players can give everyone a maximum score, and *scarcity *(*S* from now on) where a fixed budget of scores could be distributed. Direct benefit for reputation is manipulated as reputation scores are either symbolic (*not paid, NP*) or incentivized financially (*paid well, PW*). We expect that the impact of our manipulations will not be independent of each other. We predict that the highest cooperation level will appear under the condition when individuals are managing scarce resources, while the lowest will happen in a monetarily incentivized context where the evaluation of partners is not relative and therefore competition is less intense. The schematic representation of our experimental design is displayed in Fig. 2.

## Results

### Cooperation

**Figure 3:**
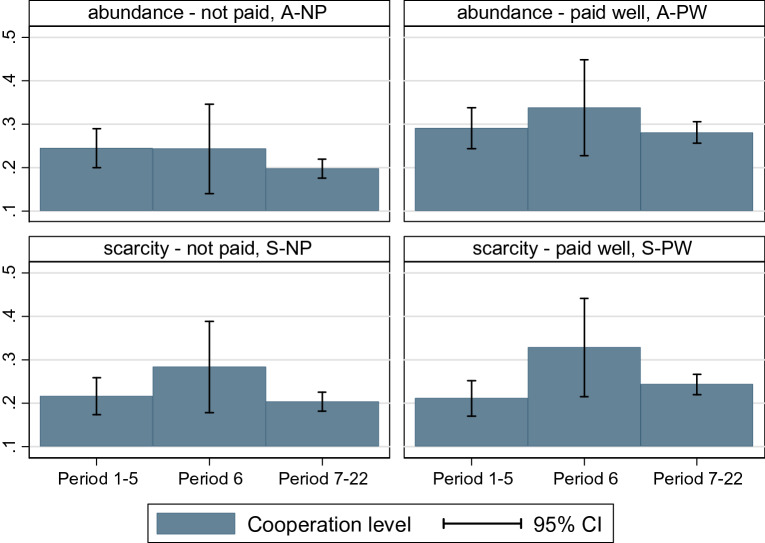
Average cooperation level before (Round 1–5), after (Round 7–22) and at the time when manipulations were introduced (Round 6) by treatments (see abbreviations above each bar chart: *A-NP* abundance, not paid; *A-PW* abundance, paid well; *S-NP* scarce, not paid; *S-PW* scarce, paid-well). The level of cooperation increased in three of the treatments, but the difference is significant in one case only (S-PW). Over time, in each treatment, cooperation fell back to or below the initial level, which is a typical finding in Prisoner’s Dilemma experiments^43^.

The introduction of gossip temporarily increased the level of cooperation in three of the four treatment conditions (A-PW: 33.8%, S-NP: 28.4%, S-PW: 32.9%) compared to the first five rounds where communication was not allowed (A-PW: 29.1%, S-NP: 21.6%, S-PW: 21.1%). Cooperation did not increase in the A-NP treatment (Round 6: 24.3%, Round 1–5: 24.5%, Fig. 3). Inspecting how decisive the changes are, we run multilevel logistic regression analysis, which revealed that the possibility of information exchange increased cooperation significantly only in the S-PW treatment (β = 0.8665 *p* < 0.01 see Supplementary Table S1, in A2), which means only a partial confirmation of H1. Neither manipulation alone had enough positive effect to result in significantly different cooperation in the long run (see Round 7–22 effects in Supplementary Table S1, in A2). Overall, cooperation was highest in the A-PW condition (Fig. 3), because the baseline cooperation was higher.

### Gossip

**Figure 4:**
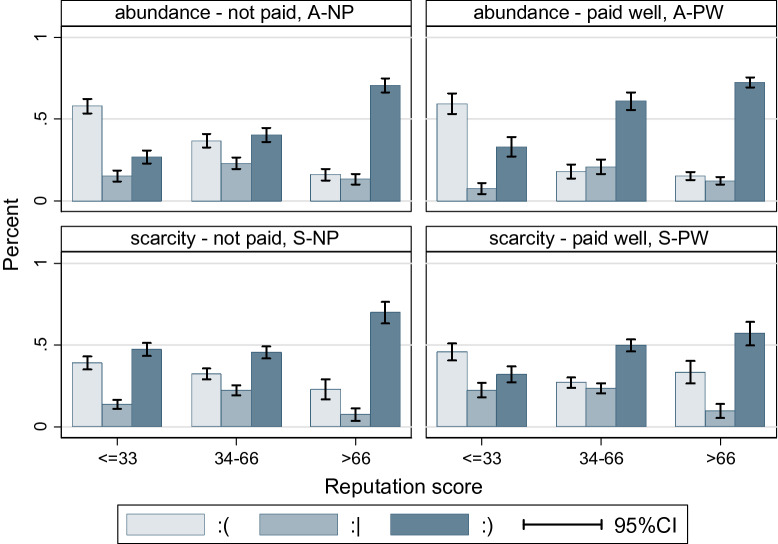
The distribution of gossip sent and its relation to reputation scores. This figure compares the proportions (*y*-axis) of positive, neutral and negative gossip among individuals with low, medium and high reputation scores (*x*-axis). Scores in players’ private reputation tables were categorized into three groups (≤ 33, 34–66, > 66). Bars shows the distribution of the valence of gossip (light blue: negative, mid blue: neutral, dark blue: positive). A breakdown by reputation score of the gossip targets shows weaker relation in S-PW between gossip targets’ trust scores and the valence of gossip. Here negative gossip is more prevalent among participants with high scores, than in other treatments.

In the experiment, participants could send three types of messages about the selected target: a happy, a neutral, or a sad smiley. Only 52% of the possible messages were sent by the participants (A-NP: 53.5%, A-PW: 51.8%, S-NP: 56.8%, S-PW: 45.7%). Positive gossip was more prevalent in the A-PW treatment (see Fig. 4 and ANOVA tables in Supplementary Table S2, S3, in A2).

The valence of gossip was very much in line with observed choices (H1a). A positive message was more likely to be sent if someone was cooperative (Ego cooperate–Alter cooperate: β = 3.3595 *p* < 0.001, Ego defect–Alter cooperate: β = 2.4422 *p* < 0.001), and a negative message if someone did not cooperate (Ego cooperate–Alter defect: β = − 2.7489 *p* < 0.001, Ego defect–Alter defect: β = − 0.9184 *p* < 0.001 see Model 2, Supplementary Table S5, in A2). Related to this effect, we found slight differences in the A-PW treatment compared to other treatment groups. On the one hand, cooperators here sent negative gossip about defectors to a smaller extent (β = 0.9974 *p* < 0.05 see A-PW × ego cooperate–alter defect interaction effect in Model 3, Supplementary Table S5, in A2). On the other hand, gossip about observed cooperators in the A-PW treatment condition was less positive in comparison to other conditions (β = − 0.8523 *p* < 0.05, see A-PW × alter cooperate interaction effect in Model 3, Supplementary Table S5 in A2).

Higher reputational points increased the probability of more positive messages (β = 0.0208 *p* < 0.001, see the effect of reputation score distributed to alter in the previous round in Model 2, Supplementary Table S5, in A2). The S-PW treatment modifies the effect of reputational position on gossip: in the presence of competition with monetary rewards strategically motivated gossip is more prevalent as the evaluation of individuals with high reputation is more negative (β = − 0.0095 *p* < 0.05, see S-PW × reputation score distributed to alter in the previous round interaction effect in Model 3, Supplementary Table S5, in A2).

### Reputation

**Figure 5:**
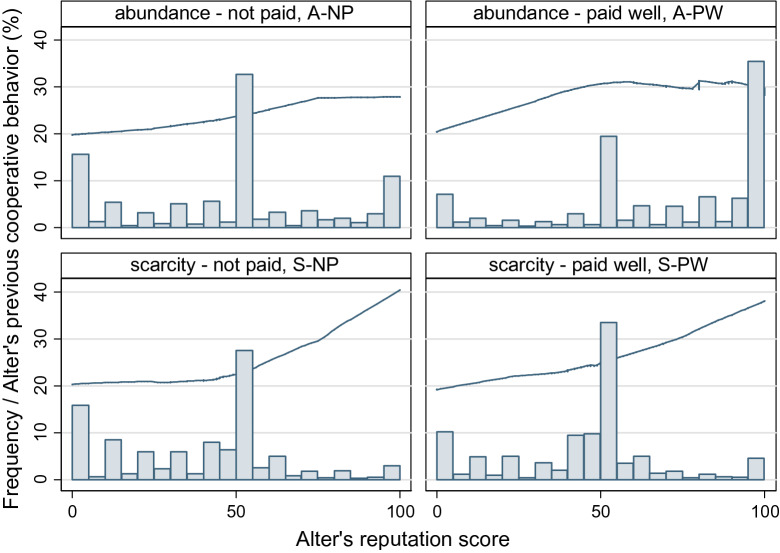
The distribution of reputation scores and its accuracy. Treatment groups without competition have been characterized by higher scores, and presence of lower scores was more typical in ‘not paid’ treatments. These trends observed along the two manipulations causes the difference in the distribution of reputation scores between the four treatments: A-PW was characterized by merely high scores and the S-NP by rather low scores. In sessions A-NP we see a wide-spread scoring in both low and high directions, while in S-PW we observe a less extreme negative shift in the reputation score distribution. A LOWESS fitted line shows how well reputation scores reflect past behaviours. Scarcity of reputational scores better distinguishes cooperative individuals.

The average reputation score players gave to each other is slightly lower than the initial value of 50 (M = 48.5, SD = 30.3), and the way reputation scores were distributed among participants varies in the different treatments (see Fig. 5). Higher scores are more frequent in treatment group A-PW (M = 68.4, SD = 32.0), where monetary incentives were used and participants could give high scores without lowering the scores of other players (see ANOVA tables in Supplementary Tables S6, S7, in A2).

We estimated a multilevel linear regression model to explain the allocation of reputation scores. We found that the cooperative decisions of interaction partners (Ego cooperate–Alter cooperate: β = 16.0085 *p* < 0.001, Ego defect–Alter cooperate: β = 8.7767 *p* < 0.001) and observed players (β = 4.2662 *p* < 0.001), as well as positive messages (β = 7.1155 *p* < 0.001) had a significant positive effect on the allocated reputation scores, while defections (Ego cooperate–Alter defect: β = − 12.3325 *p* < 0.001, Ego defect–Alter defect:β = − 2.9128 *p* < 0.001, observed Alter defect: β = − 1.3760 *p* < 0.001) and negative messages (β = − 6.9627 *p* < 0.001) negatively influenced scores (H1b) (see Model 2, Supplementary Table S9, in A2). More messages were rewarded with higher reputation scores (see Model 2, Supplementary Table S9, in A2), but only in S-PW (Nr. of gossip sent by Alter–treatment interaction: β = 0.8291 *p* < 0.05 in Model 4, Supplementary Table S9, in A2). Looking for further differences between the treatments (see gossip–treatment interaction effect in Model 3, Supplementary Table S9, in A2) we found that in A-PW negative and neutral gossip generated a greater volume of score reduction (negative gossip: β = − 5.5211 *p* < 0.001, neutral gossip: β = − 5.5822, *p* < 0.001) and positive gossip was more powerful (β = 5.7895 *p* < 0.001) in S-PW in comparison to other treatments.

Information from trustworthy sources might affect how much individuals rely on them. In this experiment the identity of the gossip partner was known, therefore players might have stored gossip differently when a randomly selected gossip partner had higher scores in their private reputation table. This assumption is twofold: an increase in the reputation of the gossip sender implied higher score reduction in case of negative gossip, while positive gossip causes a smaller raise if the sender is more trustworthy (see Gossip Partner’s reputation–Gossip interaction effect in models for each treatment in Supplementary Table S10, in A2).

### Social norms

While social norms in IR models are conceptualized as expectations on public reputation, in our experiment we are able to explore the presence of shared properties of the leading eight social norms after privately observed actions for privately assigned reputations. Multilevel mixed-effect linear regressions were used, where the dependent variable was the reputation score that has been allocated to other participants. Predicted values of updated reputations derived from the model are summarized in Table 1, Panel B (see detailed results in Model 1 in Supplementary Table S11, in A2). We do not observe changes between bad and good reputation, if we assume the default value of 50 to be the neutral point. Therefore, results should be interpreted in conjunction with predicted changes (see in Table 1, Panel C, details in Model 2 in Supplementary Table S11, in A2).

**Table 1:**
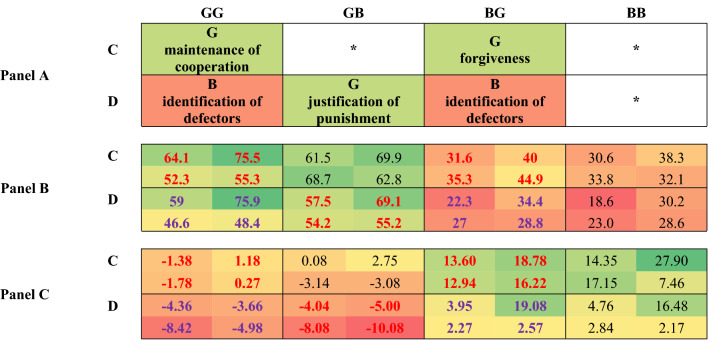
Means of predicted reputation scores (Panel B) and mean of predicted changes in reputation scores (Panel C) of the focal player after the observation of a play between the focal player and his opponent in our experiment. Rows show the action of the focal player (C: cooperate, D: defect). Columns show the potential combinations of reputation scores for the focal player (first letter) and the opponent (second letter) (G: good, B: bad). Results can be compared to the table of social norms^19^. Expected common properties of the leading eight norms are indicated with red (good) and purple (bad) font. Each cell contains predicted reputation scores divided by treatment condition (upper-left: A-NP, upper-right: A-PW bottom-left: S-NP, bottom-right: S-PW).

There are four main features of leading eight norms^19^: (i) maintenance of cooperation, (ii) identification of defectors, (iii) justification of punishment and (iv) forgiveness (see Table 1, Panel A). We confirm the existence of some of these conditions of the leading eight social norms, however some others are missing. (i) The “*maintenance of cooperation*” corresponds to the characteristic that cooperation between good parties upholds good reputation (C-GG in Table 1, Panel B and C). This feature seems to be present in our sample. (ii) “*Identification of defectors*” concerns both good and bad focal players and exists if defection against a good co-player leads to bad reputation. (iii) “*justification of punishment*” appears to be present, because despite defection good reputation remains good (D-GB in Table 1, Panel B). Changes, however, demonstrate a negative shift in reputation (D-GB in Table 1, Panel C), which contradicts the notion. (iv) Finally, “f*orgiveness”*: we cannot observe a change from bad to good reputation (C-BG in Table 1, Panel B), however, cooperative acts improve bad players’ reputation (C-BG in Table 1, Panel C). The average reputation score of good focal players after such defection falls below 50 only in scarcity treatments (GG-D in Table 1, Panel B, only S-NP is significantly different β = − 0.0023 p < 0.05, see detailed treatment differences in Model 2 in Supplementary Table S11, in A2).

All in all, the predicted reputational scores are in line with the leading eight norms in the conditions: maintenance of cooperation, justification of punishment, and identification of defectors in case of bad donors, however they contradict the leading eight norms in the conditions of forgiveness and identification of defectors in case of good donors. However, the predicted change of reputational scores are in line with the leading eight norms in the conditions: forgiveness and identification of defectors in case of good donors. In other words, the absolute scores and predicted change always oppose each other. When the absolute score fits the predictions of the leading eight the predicted change does not and vice versa.

We found two outstanding effects behind these outcomes. The most important was that the focal player’s action, which is considered in first order social norms, significantly contributed to the focal player’s reputation (β = 12.2038 p < 0.001 in Model 1 in Supplementary Table S11, in A2). The second obviously strong effect—as colours of Table 1 indicate—is the reputation of the focal player. These results show a clear effect of first order social norms but leave uncertainties about the functioning of higher order norms.

### Positive assortment

A reputation system is reliable if it reflects past behaviour of others. Using aggregate statistics, we found small, but significant correlations between the level of cooperation against someone and their overall cooperativeness only in S-PW treatment (*ρ* = 0.38, *p* < 0.05) if all periods are considered (see Supplementary Table S12, in A2). Taking into account later periods the same correlation came into sight from Round 10 in A-NP (*ρ* = 0.32, *p* < 0.05) and correlation in S-PW became higher (Round 10–22: 0.42, *p* < 0.01). Using multilevel logistic regression models, we verified that the reputation system improved its credibility in the A-NP (β = 1.1662 *p* < 0.001) and S-PW (β = 0.9393 *p* < 0.01) treatments over time (see Alter’s previous cooperative behaviour effect in Model 1, Supplementary Table S13, in A2). Partners’ reputation positively influenced decision making in each treatment, but in A-PW reputation scores have lower effect on cooperation (β = − 0.0278 *p* < 0.001, see A-PW alter’s reputation score interaction effect in Joint Model, Supplementary Table S13, in A2).

**Figure 6:**
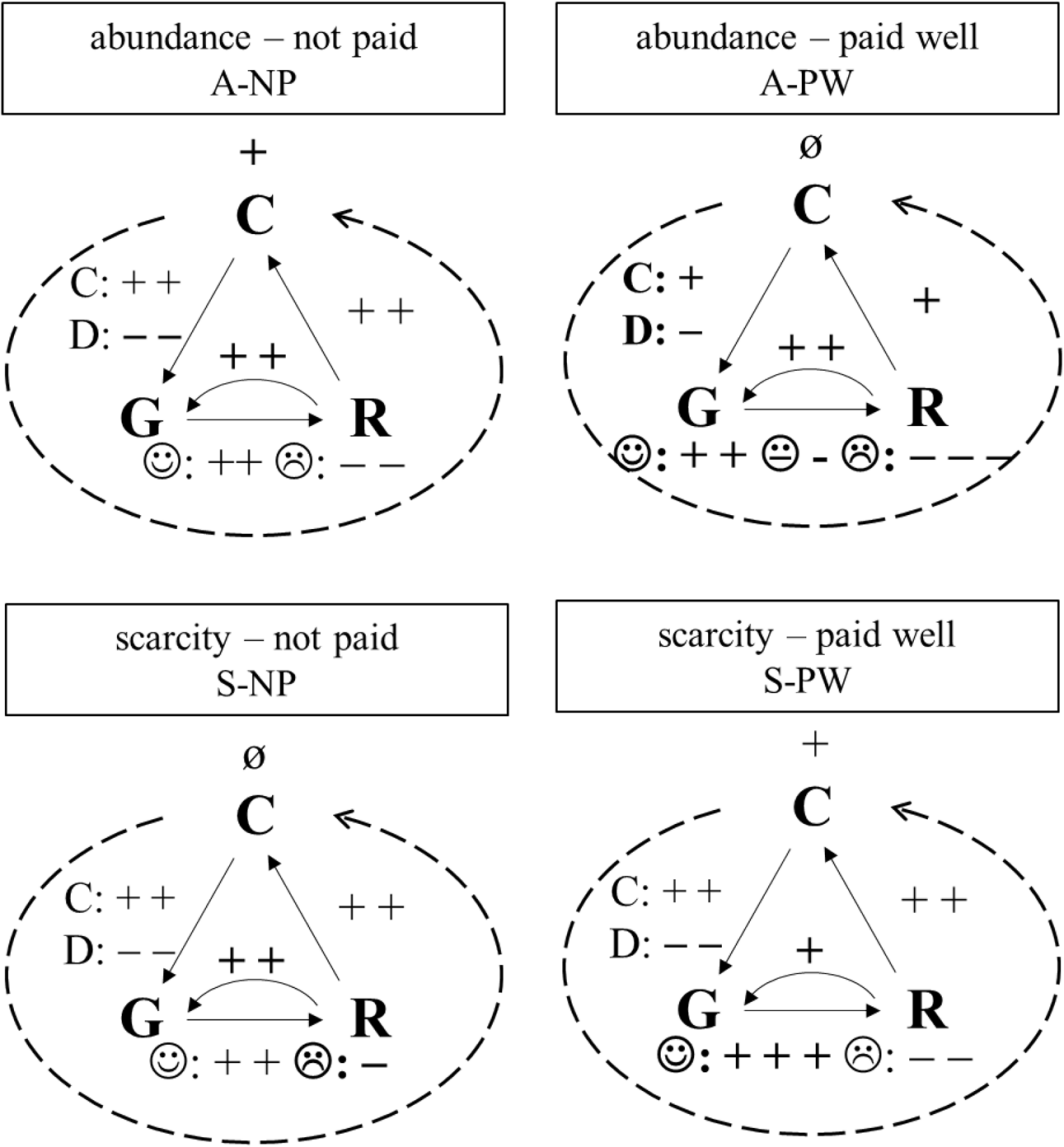
A summary of our results. In the figure, C, G, and R represent cooperation, gossip, and reputation respectively. The left side of the triangle shows how gossip was influenced by participants’ PD choices (*C *cooperation, *D *defection). The lower side of the triangle outlines how participants change reputation scores as a result of positive (:)) and negative (:() gossip. The right arrow represents the use of reputation scores in decision making. The relative strength of effects clarifies the distinction between treatments. The circle arrow (with its effect at the top) shows the overall accuracy of the reputation system.

We summarized our results in Fig. 6, where the reader can follow each step of the reputation mechanism and differences between treatments. Curved arrows represent positive assortment and signs at the top of these arrows indicate the success of reputational information transmission. The association that individuals collaborate with individuals who have previously cooperated with others takes place under two conditions: external incentives with competition (S-PW) and internal rewards (cooperation) with universal access (A-NP). Successful mapping of individuals’ willingness to cooperate in A-PW was hindered by the fact that cooperative third-party observations were not rewarded here with positive gossip as much as in other conditions and defectors were less punished with negative gossip. This leniency was somewhat counterbalanced as a negative gossip was followed by stronger point reduction. In S-NP, we observe reverse behaviour: negative gossip has a smaller effect on reputation. It is interesting to note that participants overrate positive gossip in S-PW. The last arrow of the triangle shows weaker reputation-based cooperation in A-PW.

## Discussion

We investigated how cooperation can be sustained by private reputations formed by direct observations and gossip. We found a slight increase in the level of cooperation in one condition when the institution of reputations and gossip have been introduced (H1). Cooperation has faded over time, which is a typical feature of Social Dilemma experiments^76^. As preconditions of gossip to be effective, we expected and confirmed that gossip was in line with previous cooperation choices (H1a) and gossip received has altered the private reputations of others (H1b).

We proposed two mechanisms that could safeguard the credibility of gossip for informing the choice of cooperation. First, we investigated whether the scarcity of reputational resources (H2) with the expectation that reduced access will increase competition, could increase reputation-based cooperation. Second, we investigated whether additional monetary incentives connected to reputation would distort the credibility of reputations (H3). We found that neither the scarcity of reputational scores nor monetary incentives alone could maintain reputation-based cooperation on the long term. We showed, however, that at the intersections of these two manipulations, competition for scarce monetary rewards resulted in higher cooperation in the short run. To better understand our results, we discuss each treatment in detail in Supplementary Material A3.

A reputational system is reliable if it appropriately reflects the potential behaviour of others that is otherwise hidden to new partners. It functions well if it helps individuals to cooperate with those who have a higher reputation and defect against those who have lower willingness to cooperate. Our results suggest that motivation for building a proper reputation system increases if people find it easy (without competition) to credibly signal prosociality (non-monetary rewards) or if external incentives encourage everyone to participate in the competition—maybe because higher positions in the reputation hierarchy are more robust. Even though reputations have seemingly been well translated to gossip under these conditions, they did not increase strategic cooperation in a long run in the one-shot PD game with stranger matching.

We found that gossip was influenced by previous reputational scores not just by the last observed action. Since reputation scores are influenced by messages beyond actions, reputation scores could have been inflated in the informal communication process. Hence, positive gossip increased good reputations and negative gossip downgraded bad reputations. This has important implications for the whole dynamics of the development or the maintenance of reputational systems.

The analysis of social norms has revealed a similar effect: while the strongest predictor of reputational scores was the focal actor’s behaviour (cooperation vs. defection), the previous reputation score also had a significant impact. In other words, there was a strong inheritance of reputational scores. Reputation updates were influenced by actions and previous reputations, but in little alignment with the “leading eight” social norms^19^. Most importantly, the reputation of the opponent had a little effect on the update of reputation of the focal player (see Table 1, Panel C). This has two important implications: both justified punishment and the identification of defectors might be missing from the system, i.e., defection against an opponent in good or bad state has very similar effects. While the scope of the investigation of higher order norms is limited in our experiment, yet it shows that the presence of leading eight social norms cannot be taken for granted.

The combined effect of the reputation of the focal player both on gossip and reputational updating could explain the lack of increase of cooperation in our model on the long term. Beyond the ineffectiveness of the examined reputation systems, the fact that we do not experience a larger impact of reputations on cooperation could be attributed to several other factors. Primarily, we investigated the two-person PD game with random reshuffling of partners and no publicly available information, which itself is the most severe social dilemma in which rational action is simply defection. The magnitude of conflict in the Prisoner’s Dilemma game could be so strong that even a well-functioning reputation system could not increase cooperative acts^62^. Unfortunately, in this study it is impossible to assess whether the magnitude of the conflict is responsible for the low level of cooperation. The PD game is an interdependent situation and hence it is not the most appropriate to test fundamental tenets of the theory of competitive altruism^48–50^. Future studies could investigate if scarce reputations and direct reputational incentives could increase giving in the dictator game in the lab or in field settings. In our experiment, cooperation could have collapsed before the reputation system had been sufficiently developed, which leaves open the question of the coevolution of reputation and cooperation^77^. It is also possible that reputation scores worked to a limited extent because they were not directly communicated to others or due to the abstract situation and scores in the experiment. Even more, participants potentially had problems to remember their earlier experiences and might have also mixed up other participants as they were identified with numbers that are harder to recall than names or faces. We should also caution about the direct correspondence of our study to the theoretical literature as we used private reputations that are realistic but contradict to the assumption of publicly available and perfect information on choices or reputations in showcased models of cooperation^10,11,18–20^. As reputations were private in our experiment, they could be used only to a limited extent for strategic reasons, and they could be linked to cooperation only through simplified gossip communication. This also limits the connection of our study to the theory of competitive altruism^48–50^ as privately stored reputations cannot be used by the recipients for acquiring diverse benefits such as status, power, or access to resources, and participants could not select their interaction partners^78,79^.

Still, our results bring us closer to understanding under which conditions reputations and gossip contribute to cooperation. Further research is needed to find out under which conditions gossip is used strategically and in a dishonest way to undermine the reputation of others, and under which conditions it could be considered as altruistic punishment^80^.

## Methods

### Participants

We investigated our hypotheses in an experimental computer laboratory with volunteer participants. The experiment was conducted at the Corvinus University of Budapest between 13–25 November 2016. In total, 160 individuals (46% female, 23.2 years old on average) participated in the experiment (male: 54.4%) in eight sessions in groups of 20. The final profit was calculated as an average payoff of 6 randomly selected rounds. In addition to the final payoff, a show-up fee (HUF 1000) has been paid to the participants. Participants earned 1822 HUF on average.

### Procedure

The experiment has been programmed using the experimental software z-Tree^81^. Participants have read the instructions on paper and on their screen after they have been randomly assigned to a computer in the lab. Subsequently, they had to fill in a quiz of understanding and when in doubt, could ask questions privately. In the experiment, participants were identified with ID numbers ranging from 1 to 20. The experiment has been divided into two phases. Phase I took place in the first five rounds (Rounds 1–5) and Phase II run for seventeen rounds (Rounds 6–22) until the end of the experiment. Subjects had no information on the total number of rounds of the experiment, which was slightly different due to time restrictions. To consider all conditions equally in the analysis we only used 22 rounds, because which corresponds to the shortest experimental session. In the second phase, players received additional instructions. In both phases, each round began with two, simultaneously played two-person Prisoner’s Dilemma (PD) games. PD partners were randomly matched and IDs of the two opponents were displayed on participants’ screen (please see original screens in Supplementary A5 for Phase I and A6 for Phase II). PD options were labelled with ‘L’ and ‘R’. The cooperative decision was marked with ’L’. PD payoffs through the experiment were fixed to HUF 1500 (EUR 4.7) for mutual cooperation; HUF 500 (EUR 1.6) for mutual defection; HUF 2500 (EUR 7.8) for temptation; and 0 otherwise. Subjects had 23 s to decide. Results appeared on the screen after every PD game. This has completed one round in Phase I.

Rounds in Phase II were expanded with new elements of reputation and gossip. In the following, we describe these new elements in the temporal order in which they occurred on participants’ screens in each round. As the first new element, on the first screen, in addition to the PD game, a ‘reputation table’ appeared with the IDs of all other 19 players. Next to each ID, a reputation score of 50 was displayed in Round 6. Participants were told that a value of 50 was the initial value for everyone. In later rounds, privately given reputation scores from the previous round were displayed in read-only mode. After playing the two PD games, participants were informed about their payoffs in the PD games. On the second screen where PD results were displayed, as a second new element of Phase II, the IDs and choices of two other participants in one randomly selected PD were displayed. Hence, participants were able to observe the PD decisions of four players in total: of their own interaction partners in the PD games and of the two matched partners from a randomly selected game.

On the third screen, the next novelty of Phase II was introduced. Participants could send a maximum of four gossip to a randomly selected gossip partner (receiver), whose ID has appeared on their screen. Participants could enter up to four IDs of other participants (targets) in empty boxes on their screen of whom they wanted to send a message about. We limited the gossip opportunities to four possible targets as in each round, participants could observe the decisions of four other players. It was, however, not required to send gossip about these participants, since boxes could have been filled in with any ID. Participants were assisted in their gossip choices by the read-only display of their ‘reputation table’ on their screen. For each target, participants could select positive, neutral, or negative emoticons as the gossip message. Sending gossip was optional, and it was free of charge.

Gossip messages were not anonymous. On the fourth screen, incoming gossip messages became readable along with the ID of the sender. On the same screen, participants could assign or update reputation scores to all other (19) participants. More precisely, the instruction on the screen asked participants to privately evaluate how trustable they think others are on a scale of 0 to 100. Reputation scores were private assessments and participants were informed that the scores they gave to others were only visible to them. Previously given scores were displayed as reference values. On the fifth and last screen of each round, participants learned their own average score received from everyone else along with their payoff in the given round.

### Design

The experiment in Phase II followed a 2 × 2 between-subject design. Four treatment conditions were constructed by the combination of two manipulations, both of which addressed the private reputational component of the experiment. First, we manipulated the scarcity of reputational rewards, second, we modified whether reputation had direct monetary effect on participants’ payoffs. The scarcity of reputations was manipulated by the way participants could distribute reputation scores to others (on a scale between 0 and 100). Participants could either had a fixed budget of reputation scores (scarcity) or there was no ceiling on the distributable scores (abundance). In the abundance treatment, a participant could assign any number between 0 and 100 to each participant, a maximum of 1900 points was distributable (100N − 1) in total. Theoretically, it can happen that everyone achieves a maximum reputation of 100. In the scarcity treatment, we limited the distributable scores to 950 (50N − 1). If a subject here wanted to give 100 points to someone, then only 850 points have remained to be shared among the other 18 participants. Direct benefits for reputation was manipulated as reputation scores were either symbolic (not paid) or were incentivized financially (paid well). In the latter case, participants received the payoffs from the PD games and nothing more or less if they received 50 reputation points (the midscale value) from other participants on average. Otherwise, a one-unit decrease/increase from the default value of 50, reduced/increased their payments by HUF 20 (EUR 0.06). For instance, if all participants gave zero reputation to someone, then the receiver’s payment was decreased by HUF 1000 (EUR 3.12). The four experimental groups are the combination of these two manipulations. In one of the condition reputations were not paid, and individuals could obtain reputation scores as many as they want out of the 100 (*abundance-not paid*). In the second case, accessible reputation was unlimited as in the previous condition, but payoffs were affected by players’ average reputation scores (*abundance-paid well*). When unpaid reputation was scarce players reputation might have been undermined if others obtained more reputation than 50 (*scarcity-not paid*). Under the condition with limited access participants not just might ended up with bad reputation but they also paid fine because of it (*scarcity-paid well*).

### Ethics

Research was approved by the Ethics Review Committee of the Centre for Social Sciences (TK CSS). We confirm that all methods were carried out in accordance with relevant guidelines and regulations. Informed consent was obtained from all participants.

## Supplementary information

Supplementary information

## Data Availability

The data that support the findings of this study are available as Supplementary Material.
