# Supplementary material for: Scarce and directly beneficial reputations support cooperation
Source: Sci Rep. 2020 Jul 13;10:11486. doi: 10.1038/s41598-020-68123-x (PMC7359363; doi:10.1038/s41598-020-68123-x)
Supplement: Supplementary file 1 — Supplementary information [file 41598_2020_68123_MOESM1_ESM.doc]

**Scarce and directly beneficial reputations support cooperation**

# Supplementary information

# Flóra Samua,b,c,*, Szabolcs Számadód,c,e, and Károly Takácsa,c

^a^ Linköping University, The Institute for Analytical Sociology, 601 74 Norrköping, Sweden

^b^ Corvinus University of Budapest, Doctoral School of Sociology, 1018 Fővám tér 8. Budapest, Hungary

^c^ Centre for Social Sciences (TK CSS) ‘Lendület’ Research Center for Educational and Network Studies (CSS-RECENS), Budapest, 1097 Tóth Kálmán u. 4, Hungary

^d^ Department of Sociology and Communication, Budapest University of Technology and Economics, Budapest, Egry

J. u. 1. 1111, Hungary

^e^ Evolutionary Systems Research Group, Centre for Ecological Research, Klebelsberg Kuno u. 3, Tihany 8237,

Hungary

^*^Correspondence and requests for materials should be addressed to F.S. E-mail: [flora.samu@liu.se](mailto:flora.samu@liu.se)

# A1.

**Table S1. Categorization of p**otential explanations behind honest and dishonest gossip.

|  | Honest | Dishonest |
| --- | --- | --- |
| Positive | Shared interest  Strong reciprocity – reward for cooperators | Nepotism, strategic alliance, group-serving |
| Negative | Strong reciprocity – punishment of defectors | Conflict of interest – hurting rivals |

# A2.

**Table S1 Estimated coefficients with multilevel mixed-effects logistic regressions (separate models for treatments)**

| **Dependent variable: Prisoner's Dilemma choice**  **0 = defect, 1 = cooperate** | **ABUNDANCE – NOT PAID**  **(A – NP)** | **ABUNDANCE –PAID WELL**  **(A –PW)** | **SCARCITY – NOT PAID**  **(S – NP)** | **SCARCITY- PAID WELL**  **(S –PW)** |
| --- | --- | --- | --- | --- |
| baseline: Round 1-5 |  |  |  |  |
| Round 6 | 0.1518 | 0.4268 | 0.4132 | 0.8665** |
|  | [0.3552] | [0.3131] | [0.3570] | [0.3358] |
| Round 7-22 | -0.3691* | -0.0514 | -0.143 | 0.229 |
|  | [0.1622] | [0.1552] | [0.1758] | [0.1604] |
| constant | -1.6697*** | -1.3349*** | -2.3969*** | -2.0165*** |
|  | [0.3070] | [0.3054] | [0.4578] | [0.3346] |
| lns1_1_1 | 0.4875** | 0.5104*** | 0.8784*** | 0.5807*** |
|  | [0.1487] | [0.1444] | [0.1678] | [0.1542] |
| Nr. of groups | 40 | 40 | 40 | 40 |
| N of obs. | 1700 | 1700 | 1700 | 1718 |

Note: * p<.05; ** p<.01; *** p<.001, standard errors are in brackets, lns1_1_1: random intercept variance between subjects

# Table S2 Analysis of Variance of Gossip between treatments

| Source | SS | df | MS | F | Prob > F |
| --- | --- | --- | --- | --- | --- |
| Between groups | 86.28 | 3 | 28.76 | 36.88 | 0 |
| Within groups | 4403.92 | 5648 | 0.78 |  |  |
| Total | 4490.19 | 5651 | 0.79 |  |  |

*Note:* Bartlett's test for equal variances: χ2(3) = 8.7147 Prob>χ2 = 0.033

# Table S3 Comparison of Gossip by treatment (Bonferroni)

|  | ABUNDANCE - NOT PAID  (A–NP) | ABUNDANCE - PAID WELL  (A–PW) | SCARCITY - NOT PAID  (S–NP) |
| --- | --- | --- | --- |
| ABUNDANCE - PAID WELL (A–PW) | 0.32 |  |  |
|  | 0 |  |  |
| SCARCITY - NOT PAID (S–NP) | 0.08 | -0.24 |  |
|  | 0.09 | 0 |  |
| SCARCITY - PAID WELL (S–PW) | 0.05 | -0.27 | -0.03 |
|  | 0.846 | 0 | 1 |

# Table S4 Summary of Gossip by treatment

|  | Mean | Std. Dev. |
| --- | --- | --- |
| ABUNDANCE - NOT PAID (A–NP) | 2.07 | 0.91 |
| ABUNDANCE - PAID WELL (A–PW) | 2.39 | 0.84 |
| SCARCITY - NOT PAID (S–NP) | 2.15 | 0.90 |
| SCARCITY - PAID WELL (S–PW) | 2.12 | 0.88 |
| Total | 2.19 | 0.89 |

**Figure S1 Gossip values by PD outcomes and treatment**

**Table S5 Random-effe**cts ordered logistic models

| Dependent variable: Ego’s gossip choice about Alter  1 = :( 2 = :| 3 = :) | **Model 1** | **Model 2** | **Model 3** |
| --- | --- | --- | --- |
| baseline: ABUNDANCE - NOT PAID (A–NP) |  |  |  |
| ABUNDANCE - PAID WELL (A–PW) | 0.9570** | 0.6639 | 0.7738 |
|  | [0.3615] | [0.3951] | [0.4720] |
| SCARCITY - NOT PAID (S–NP) | 0.2038 | 0.4114 | 0.3218 |
|  | [0.3600] | [0.3923] | [0.4480] |
| SCARCITY - PAID WELL (S–PW) | 0.2183 | 0.1435 | 0.472 |
|  | [0.3620] | [0.3950] | [0.4632] |
| PD decision (played)  baseline: Not a PD Partner |  |  |  |
| Ego cooperate – Alter cooperate |  | 3.3595*** | 3.6162*** |
|  |  | [0.2915] | [0.6533] |
| Ego cooperate – Alter defect |  | -2.7489*** | -3.2148*** |
|  |  | [0.1603] | [0.3288] |
| Ego defect – Alter cooperate |  | 2.4422*** | 2.3056*** |
|  |  | [0.1790] | [0.3094] |
| Ego defect – Alter defect |  | -0.9184*** | -0.9155*** |
|  |  | [0.0940] | [0.1756] |
| PD decision (observed)  baseline: Not observed |  |  |  |
| Alter cooperate |  | 1.3421*** | 1.3448*** |
|  |  | [0.1540] | [0.2904] |
| Alter defect |  | -0.7453*** | -0.8634*** |
|  |  | [0.0951] | [0.1851] |
| Reputation score distributed to Alter in the previous round |  | 0.0208*** | 0.0241*** |
|  |  | [0.0014] | [0.0026] |
| Round |  | -0.0034 | -0.0036 |
|  |  | [0.0075] | [0.0075] |
| PD decision (played) – treatment interaction |  |  |  |
| ABUNDANCE - PAID WELL (A–PW) * Ego cooperate – Alter cooperate |  |  | -0.9999 |
|  |  |  | [0.7865] |
| SCARCITY - NOT PAID (S–NP) * Ego cooperate – Alter cooperate |  |  | 0.9024 |
|  |  |  | [1.0648] |
| SCARCITY - PAID WELL (S–PW) * Ego cooperate – Alter cooperate |  |  | -0.0085 |
|  |  |  | [0.8805] |
| ABUNDANCE - PAID WELL (A–PW) * Ego cooperate – Alter defect |  |  | 0.9974* |
|  |  |  | [0.4312] |
| SCARCITY - NOT PAID (S–NP) * Ego cooperate – Alter defect |  |  | 0.0052 |
|  |  |  | [0.4858] |
| SCARCITY - PAID WELL (S–PW) * Ego cooperate – Alter defect |  |  | 0.5758 |
|  |  |  | [0.4740] |
| ABUNDANCE - PAID WELL (A–PW) * Ego defect – Alter cooperate |  |  | 0.7942 |
|  |  |  | [0.6395] |
| SCARCITY - NOT PAID (S–NP) * Ego defect – Alter cooperate |  |  | 0.0426 |
|  |  |  | [0.4643] |
| SCARCITY - PAID WELL (S–PW) * Ego defect – Alter cooperate |  |  | 0.1958 |
|  |  |  | [0.4604] |
| ABUNDANCE - PAID WELL (A–PW) * Ego defect – Alter defect |  |  | -0.2685 |
|  |  |  | [0.2650] |
| SCARCITY - NOT PAID (S–NP) * Ego defect – Alter defect |  |  | 0.2223 |
|  |  |  | [0.2514] |
| SCARCITY - PAID WELL (S–PW) * Ego defect – Alter defect |  |  | -0.0476 |
|  |  |  | [0.2660] |
| PD decision (observed) – treatment interaction |  |  |  |
| ABUNDANCE - PAID WELL (A–PW) * Alter cooperate |  |  | -0.8523* |
|  |  |  | [0.4217] |
| SCARCITY - NOT PAID (S–NP) * Alter cooperate |  |  | -0.027 |
|  |  |  | [0.4470] |
| SCARCITY - PAID WELL (S–PW) * Alter cooperate |  |  | 0.636 |
|  |  |  | [0.4190] |
| ABUNDANCE - PAID WELL (A–PW) * Alter defect |  |  | 0.3106 |
|  |  |  | [0.2803] |
| SCARCITY - NOT PAID (S–NP) * Alter defect |  |  | 0.2074 |
|  |  |  | [0.2574] |
| SCARCITY - PAID WELL (S–PW) * Alter defect |  |  | 0.0194 |
|  |  |  | [0.2684] |
| Reputation score distributed to Alter in the previous round – treatment interaction |  |  |  |
| ABUNDANCE - PAID WELL (A–PW) * Reputation score distributed to Alter in the previous round |  |  | -0.0033 |
|  |  |  | [0.0037] |
| SCARCITY - NOT PAID (S–NP) * Reputation score distributed to Alter in the previous round |  |  | -0.0002 |
|  |  |  | [0.0038] |
| SCARCITY - PAID WELL (S–PW) * Reputation score distributed to Alter in the previous round |  |  | -0.0095* |
|  |  |  | [0.0038] |
| cut1 | -0.7312** | -0.3212 | -0.2387 |
|  | [0.2529] | [0.3048] | [0.3368] |
| cut2 | 0.2786 | 1.0574*** | 1.1494*** |
|  | [0.2528] | [0.3052] | [0.3372] |
| sigma2_u | 2.3562*** | 2.7842*** | 2.8148*** |
|  | [0.3200] | [0.3816] | [0.3867] |
| Nr. of groups | 156 | 156 | 156 |
| Nr. of obs. | 5599 | 5480 | 5480 |

*Note*: * p<.05; ** p<.01; *** p<.001, standard errors are in brackets, sigma2_u: variance component attributable to subjects

# Table S6 Analysis of Variance of Reputation Score between treatments

| Source | SS | df | MS | F | Prob > F |
| --- | --- | --- | --- | --- | --- |
| Between groups | 7275618.90 | 3 | 2425206.30 | 3110.80 | 0 |
| Within groups | 40372794.30 | 51786 | 779.61 |  |  |
| Total | 47648413.20 | 51789 | 920.05 |  |  |

# *Note:* Bartlett's test for equal variances: chi2(3) = 1.8e+03 Prob>chi2 = 0.000

# Table S7 Comparison of Reputation Score by treatment (Bonferroni)

|  | ABUNDANCE - NOT PAID (A–NP) | ABUNDANCE - PAID WELL (A–PW) | SCARCITY - NOT PAID (S–NP) |
| --- | --- | --- | --- |
| ABUNDANCE - PAID WELL (A–PW) | 22.68 |  |  |
|  | 0 |  |  |
| SCARCITY - NOT PAID (S–NP) | -8.56 | -31.24 |  |
|  | 0 | 0 |  |
| SCARCITY - PAID WELL (S–PW) | -2.68 | -25.36 | 5.88 |
|  | 0 | 0 | 0 |

# Table S8 Summary of Reputation Score by treatment

|  | Mean | Std. Dev. |
| --- | --- | --- |
| ABUNDANCE - NOT PAID (A–NP) | 45.68 | 30.52 |
| ABUNDANCE - PAID WELL (A–PW) | 68.36 | 31.97 |
| SCARCITY - NOT PAID (S–NP) | 37.12 | 24.76 |
| SCARCITY - PAID WELL (S–PW) | 43.00 | 23.49 |
| Total | 48.53 | 30.33 |

**Table S9 Multilev**el mixed-effects linear regression

| **Dependent variable: Reputation score distributed to Alter**  **between 0 – 100** | **Model 1** | **Model 2** | **Model 3** | **Model 4** |
| --- | --- | --- | --- | --- |
| baseline: ABUNDANCE - NOT PAID (A–NP) |  |  |  |  |
| ABUNDANCE - PAID WELL (A–PW) | 22.6828*** | 6.7665*** | 6.9360*** | 6.9257*** |
|  | [3.5550] | [1.1243] | [1.1258] | [1.1265] |
| SCARCITY - NOT PAID (S–NP) | -8.5606* | -2.6015* | -2.7537* | -2.8260* |
|  | [3.5550] | [1.1227] | [1.1243] | [1.1249] |
| SCARCITY - PAID WELL (S–PW) | -2.6835 | -0.9127 | -1.2385 | -1.3329 |
|  | [3.5550] | [1.1225] | [1.1238] | [1.1244] |
| baseline: Not a PD Partner |  |  |  |  |
| Ego cooperate – Alter cooperate |  | 16.0085*** | 16.0042*** | 15.9881*** |
|  |  | [0.8419] | [0.8410] | [0.8409] |
| Ego cooperate – Alter defect |  | -12.3325*** | -12.3404*** | -12.3412*** |
|  |  | [0.5039] | [0.5033] | [0.5033] |
| Ego defect – Alter cooperate |  | 8.7767*** | 8.8255*** | 8.8222*** |
|  |  | [0.5353] | [0.5347] | [0.5347] |
| Ego defect – Alter defect |  | -2.9128*** | -2.8934*** | -2.8946*** |
|  |  |  |  |  |
| baseline: Not observed |  |  |  |  |
| Alter cooperate |  | 4.2662*** | 4.3118*** | 4.3009*** |
|  |  | [0.4490] | [0.4486] | [0.4485] |
| Alter defect |  | -1.3760*** | -1.3698*** | -1.3734*** |
|  |  | [0.2556] | [0.2554] | [0.2554] |
| Reputation score distributed to Alter in the previous round |  | 0.7325*** | 0.7327*** | 0.7328*** |
|  |  | [0.0030] | [0.0030] | [0.0030] |
| Round |  | 0.0061 | 0.0069 | 0.0066 |
|  |  | [0.0141] | [0.0140] | [0.0140] |
| baseline: No gossip |  |  |  |  |
| :( |  | -6.9627*** | -7.0779*** | -7.1236*** |
|  |  | [0.3776] | [0.6896] | [0.6900] |
| :| |  | -1.7248*** | -0.854 | -0.9002 |
|  |  | [0.5144] | [1.0018] | [1.0021] |
| :) |  | 7.1155*** | 5.3406*** | 5.2942*** |
|  |  | [0.3036] | [0.6318] | [0.6323] |
| Nr. of gossip sent by Alter |  | 0.3140** | 0.3117** | -0.0409 |
|  |  | [0.1171] | [0.1170] | [0.2320] |
| Gossip – treatment interaction |  |  |  |  |
| :( * ABUNDANCE - PAID WELL (A–PW) |  |  | -5.5211*** | -5.5107*** |
|  |  |  | [1.1143] | [1.1150] |
| :( * SCARCITY - NOT PAID (S–NP) |  |  | 2.4907* | 2.5652** |
|  |  |  | [0.9843] | [0.9851] |
| :( * SCARCITY - PAID WELL (S–PW) |  |  | 1.8458 | 1.9457 |
|  |  |  | [1.0472] | [1.0480] |
| :| * ABUNDANCE - PAID WELL (A–PW) |  |  | -5.5822*** | -5.5717*** |
|  |  |  | [1.5266] | [1.5271] |
| :| * SCARCITY - NOT PAID (S–NP) |  |  | 0.7912 | 0.8671 |
|  |  |  | [1.4066] | [1.4071] |
| :| * SCARCITY - PAID WELL (S–PW) |  |  | 0.1373 | 0.2359 |
|  |  |  | [1.4096] | [1.4101] |
| :) * ABUNDANCE - PAID WELL (A–PW) |  |  | 1.1936 | 1.2037 |
|  |  |  | [0.8351] | [0.8362] |
| :) * SCARCITY - NOT PAID (S–NP) |  |  | 0.8872 | 0.9636 |
|  |  |  | [0.8643] | [0.8653] |
| :) * SCARCITY - PAID WELL (S–PW) |  |  | 5.7895*** | 5.8885*** |
|  |  |  | [0.9253] | [0.9261] |
| Nr. of gossip sent by Alter – treatment interaction |  |  |  |  |
| ABUNDANCE - PAID WELL (A–PW) |  |  |  | 0.0692 |
|  |  |  |  | [0.3290] |
| SCARCITY - NOT PAID (S–NP) |  |  |  | 0.563 |
|  |  |  |  | [0.3199] |
| SCARCITY - PAID WELL (S–PW) |  |  |  | 0.8291* |
|  |  |  |  | [0.3436] |
| constant | 45.6831*** | 12.0364*** | 12.0885*** | 12.1354*** |
|  | [2.5138] | [0.8313] | [0.8320] | [0.8323] |
| lns1_1_1 | 2.7630*** | 1.5981*** | 1.5976*** | 1.5975*** |
|  | [0.0563] | [0.0584] | [0.0584] | [0.0584] |
| lnsig_e | 3.1350*** | 2.7503*** | 2.7491*** | 2.7490*** |
|  | [0.0031] | [0.0031] | [0.0031] | [0.0031] |
| Nr. of groups | 160 | 160 | 160 | 160 |
| Nr. of obs. | 51790 | 51790 | 51790 | 51790 |

*Note*: * p<.05; ** p<.01; *** p<.001, standard errors are in brackets, lns1_1_1: random intercept variance between subjects, lnsig_e: random intercept variance within subjects

**Table S10 Multilevel mixed-effects linear regression**

| **Dependent variable: Reputation score distributed to Alter**  **between 0 – 100** | **ABUNDANCE – NOT PAID**  **(A – NP)** | **ABUNDANCE –PAID WELL**  **(A –PW)** | **SCARCITY – NOT PAID**  **(S – NP)** | **SCARCITY- PAID WELL**  **(S –PW)** |
| --- | --- | --- | --- | --- |
| baseline: Not a PD Partner |  |  |  |  |
| Ego cooperate – Alter cooperate | 20.7174*** | 10.9406* | 32.2006*** | 12.9391** |
|  | [5.7471] | [5.1270] | [6.0023] | [4.7300] |
| Ego cooperate – Alter defect | -12.9081*** | -16.5840*** | -17.5938*** | -7.2245* |
|  | [3.2688] | [3.2400] | [3.0267] | [3.3525] |
| Ego defect – Alter cooperate | 10.8297*** | 11.7203*** | 9.3753** | 6.0602 |
|  | [3.0598] | [3.3729] | [3.2897] | [4.0470] |
| Ego defect – Alter defect | -5.0406** | -6.2372** | -0.6637 | -3.4172 |
|  | [1.7044] | [2.2551] | [1.6485] | [1.8787] |
| baseline: Not observed |  |  |  |  |
| Alter cooperate | 4.3346 | 3.4113 | 7.7952** | 0.8863 |
|  | [2.4549] | [2.6545] | [2.5551] | [2.7162] |
| Alter defect | -0.2387 | -0.3301 | -2.5172 | -2.3225 |
|  | [1.5388] | [1.7692] | [1.4013] | [1.7585] |
| Reputation score distributed to Alter in the previous round | 0.7125*** | 0.5545*** | 0.6068*** | 0.5297*** |
|  | [0.0194] | [0.0237] | [0.0214] | [0.0246] |
| Round | -0.1533 | 0.0149 | 0.2071** | -0.0517 |
|  | [0.0840] | [0.1002] | [0.0800] | [0.0960] |
| Gossip Partner’s reputation | 0.1131*** | 0.1660*** | 0.1770*** | 0.2091*** |
|  | [0.0301] | [0.0353] | [0.0350] | [0.0410] |
| baseline: No gossip |  |  |  |  |
| :( | -3.2991 | 1.8383 | 1.4254 | 3.0458 |
|  | [2.2328] | [3.4742] | [2.1424] | [2.7923] |
| :| | 1.0749 | 3.5632 | 6.4906* | -1.3369 |
|  | [2.5620] | [4.7966] | [2.5268] | [3.3972] |
| :) | 10.9064*** | 17.6405*** | 12.7418*** | 24.1751*** |
|  | [2.1246] | [2.9063] | [2.1221] | [2.5961] |
| Gossip Partner’s reputation - Gossip interaction |  |  |  |  |
| :( | -0.1006** | -0.1771*** | -0.1258** | -0.1863*** |
|  | [0.0358] | [0.0451] | [0.0433] | [0.0556] |
| :| | -0.0588 | -0.0946 | -0.1533** | 0.0246 |
|  | [0.0457] | [0.0608] | [0.0545] | [0.0682] |
| :) | -0.1394*** | -0.1102** | -0.1326** | -0.2813*** |
|  | [0.0350] | [0.0369] | [0.0418] | [0.0511] |
| Nr. of gossip sent by Alter | -0.3312 | 1.1093* | 0.8784* | 1.0476* |
|  | [0.4400] | [0.5160] | [0.4228] | [0.4671] |
| _cons | 10.8726*** | 16.3587*** | 3.2347 | 10.7976*** |
|  | [2.2455] | [2.8709] | [2.0194] | [2.3715] |
| lns1_1_1 | 1.7561*** | 1.9212*** | 1.3250*** | 1.3582*** |
|  | [0.1480] | [0.1448] | [0.1805] | [0.1720] |
| lnsig_e | 2.8789*** | 3.0607*** | 2.8745*** | 2.9488*** |
|  | [0.0155] | [0.0157] | [0.0152] | [0.0164] |
| Nr. of groups | 160 | 160 | 160 | 160 |
| Nr. of obs. | 2123 | 2087 | 2213 | 1910 |

*Note*: * p<.05; ** p<.01; *** p<.001, standard errors are in brackets, Nr. of obs.: Table contains observations where Ego received gossip, lns1_1_1: random intercept variance between subjects, lnsig_e: random intercept variance within subjects

**Figure S2. The usability of the reputation systems by treatments**

Figure A2S3 depicts the average degree of cooperation with alter as a function of alter’s average cooperation level and the distribution of cooperative behaviour during the experiment. ABUNDANCE - PAID WELL (A–PW) has the lowest number of continuous defectors and SCARCITY - NOT PAID (S–NP) has the highest. There were no continuous co-operators in SCARCITY - PAID WELL (S–PW). The line graph shows ego’s decision as a function of alter’s behaviour until that round. The highest fitted slope (using Locally Weighted Scatterplot Smoothing, LOWESS method) between alter’s previous behaviour and the decision against alter was found in SCARCITY - PAID WELL (S–PW) and ABUNDANCE - NOT PAID (A–NP), so participants made the most accurate decisions in these treatments.

**Table S11 Multilevel mixed-effects linear reg**ression

|  | Model 1 | Model 2 |
| --- | --- | --- |
| Dependent variable: | Reputation distributed to Alter  between 0 – 100 | Changes in Alter’s reputation |
| Treatment (baseline: ABUNDANCE - NOT PAID (A–NP)) |  |  |
| ABUNDANCE - PAID WELL (A–PW) | 13.2715*** | 13.3583*** |
|  | [3.2063] | [3.2132] |
| SCARCITY - NOT PAID (S–NP) | 0.2067 | 0.327 |
|  | [2.6061] | [2.6315] |
| SCARCITY - PAID WELL (S–PW) | 3.1259 | 2.9222 |
|  | [2.8193] | [2.8334] |
| Focal player’s reputation | 0.8128*** | -0.1844*** |
|  | [0.0382] | [0.0383] |
| Focal player’s reputation – treatment interaction |  |  |
| Focal player’s reputation * ABUNDANCE - PAID WELL (A–PW) | -0.1715** | -0.1707** |
|  | [0.0560] | [0.0561] |
| Focal player’s reputation * SCARCITY - NOT PAID (S–NP) | -0.0292 | -0.0335 |
|  | [0.0553] | [0.0557] |
| Focal player’s reputation * SCARCITY - PAID WELL (S–PW) | -0.1536** | -0.1576** |
|  | [0.0563] | [0.0571] |
| Opponent’s reputation | -0.0025 | 0.0005 |
|  | [0.0383] | [0.0384] |
| Opponent’s reputation – treatment interaction |  |  |
| Opponent’s reputation * ABUNDANCE - PAID WELL (A–PW) | 0.0558 | 0.0524 |
|  | [0.0562] | [0.0563] |
| Opponent’s reputation * SCARCITY - NOT PAID (S–NP) | 0.0566 | 0.0547 |
|  | [0.0523] | [0.0527] |
| Opponent’s reputation * SCARCITY - PAID WELL (S–PW) | -0.0146 | -0.0116 |
|  | [0.0558] | [0.0559] |
| Focal player’s reputation – Opponent’s reputation interaction | 0.0001 | 0.0524 |
|  | [0.0006] | [0.0563] |
| Focal player’s reputation – Opponent’s reputation – treatment interaction |  |  |
| Focal player’s reputation – Opponent’s reputation * ABUNDANCE - PAID WELL (A–PW) | -0.0001 | 0.0000 |
|  | [0.0008] | [0.0008] |
| Focal player’s reputation – Opponent’s reputation * SCARCITY - NOT PAID (S–NP) | -0.0024* | -0.0023* |
|  | [0.0010] | [0.0010] |
| Focal player’s reputation – Opponent’s reputation * SCARCITY - PAID WELL (S–PW) | 0.0011 | 0.0013 |
|  | [0.0011] | [0.0011] |
| Focal player’s decision (baseline: D) |  |  |
| C | 12.2038*** | 12.1294*** |
|  | [3.6599] | [3.6601] |
| Focal player’s decision * treatment interaction |  |  |
| C * ABUNDANCE - PAID WELL (A–PW) | 0.2514 | 0.8586 |
|  | [5.8325] | [5.8384] |
| C * SCARCITY - NOT PAID (S–NP) | 3.9982 | 4.1214 |
|  | [4.7190] | [4.7431] |
| C * SCARCITY - PAID WELL (S–PW) | -14.2599** | -14.3638** |
|  | [5.2902] | [5.3146] |
| Focal player’s decision * Focal player’s reputation interaction |  |  |
| C * Focal player’s reputation | -0.1277 | -0.1306 |
|  | [0.0790] | [0.0790] |
| Focal player’s decision - Focal player’s reputation - treatment interaction |  |  |
| C * Focal player’s reputation * ABUNDANCE - PAID WELL (A–PW) | 0.033 | 0.0231 |
|  | [0.1148] | [0.1150] |
| C * Focal player’s reputation * SCARCITY - NOT PAID (S–NP) | 0.0262 | 0.027 |
|  | [0.1025] | [0.1027] |
| C * Focal player’s reputation * SCARCITY - PAID WELL (S–PW) | 0.3065** | 0.3064** |
|  | [0.1101] | [0.1107] |
| Focal player’s decision – Opponent’s reputation interaction |  |  |
| C * Opponent’s reputation | -0.0061 | 0.0025 |
|  | [0.0793] | [0.0800] |
| Focal player’s decision – Opponent’s reputation – treatment interaction |  |  |
| C * Opponent’s reputation * ABUNDANCE - PAID WELL (A–PW) | -0.1409 | -0.1592 |
|  | [0.1148] | [0.1157] |
| C * Opponent’s reputation * SCARCITY - NOT PAID (S–NP) | -0.0512 | -0.0541 |
|  | [0.1099] | [0.1107] |
| C * Opponent’s reputation * SCARCITY - PAID WELL (S–PW) | 0.3804*** | 0.3619** |
|  | [0.1148] | [0.1155] |
| Focal player’s decision – Focal player’s reputation – Opponent’s reputation |  |  |
| C * Opponent’s reputation * Focal player’s reputation | 0.0000 | -0.0001 |
|  | [0.0012] | [0.0012] |
| Focal player’s decision – Focal player’s reputation – Opponent’s reputation – treatment interaction |  |  |
| C * Opponent’s reputation * Focal player’s reputation * ABUNDANCE - PAID WELL (A–PW) | 0.0015 | 0.0018 |
|  | [0.0016] | [0.0016] |
| C * Opponent’s reputation * Focal player’s reputation * SCARCITY - NOT PAID (S–NP) | -0.0004 | -0.0004 |
|  | [0.0019] | [0.0019] |
| C * Opponent’s reputation * Focal player’s reputation * SCARCITY - PAID WELL (S–PW) | -0.0073*** | -0.0070*** |
|  | [0.0020] | [0.0020] |
| _cons | 7.4015*** | 7.3181*** |
|  | [1.9088] | [1.9195] |
| lns1_1_1 | 1.6794*** | 1.6793*** |
|  | [0.0857] | [0.0862] |
| lnsig_e | 2.9001*** | 2.8986*** |
|  | [0.0099] | [0.0100] |
| Nr. of groups | 160 | 160 |
| Nr. of obs. | 5347 | 5254 |

*Note*: * p<.05; ** p<.01; *** p<.001, standard errors are in brackets, Nr. of obs.: Table contains observations where Ego observes a PD game as a third party, lns1_1_1: random intercept variance between subjects, lnsig_e: random intercept variance within subjects

**Figure S3. Previous reputational scores and reputation updates by treatments**

*Notes:* The Figure shows how previous reputational scores affect reputation updates by treatments. The red line is fitted by using a locally weighted regression (lowess) of changes in scores on reputation scores in the previous round.

**Table S12 Overall correlation between behaviour, gossip, reputation score by treatment**

|  |  | Alter’s reputation score |  | Degree of cooperation with Alter |  | Gossip about Alter |  |
| --- | --- | --- | --- | --- | --- | --- | --- |
| Control (Round 1-5) | Alter’s cooperation |  |  | 0.04 | 0.65 |  |  |
|  | Alter’s reputation score |  |  |  |  |  |  |
|  | Degree of cooperation with Alter |  |  |  |  |  |  |
| ABUNDANCE - NOT PAID (A–NP) | Alter’s cooperation | 0.57 | 0.00 | 0.21* | 0.19 | 0.71 | 0.00 |
|  | Alter’s reputation score |  |  | 0.55 | 0.00 | 0.74 | 0.00 |
|  | Degree of cooperation with Alter |  |  |  |  | 0.38 | 0.01 |
| ABUNDANCE - PAID WELL (A–PW) | Alter’s cooperation | 0.75 | 0.00 | 0.16 | 0.31 | 0.63 | 0.00 |
|  | Alter’s reputation score |  |  | -0.01 | 0.93 | 0.64 | 0.00 |
|  | Degree of cooperation with Alter |  |  |  |  | 0.25 | 0.13 |
| SCARCITY - NOT PAID (S–NP) | Alter’s cooperation | 0.43 | 0.01 | 0.04 | 0.83 | 0.71 | 0.00 |
|  | Alter’s reputation score |  |  | -0.29 | 0.07 | 0.64 | 0.00 |
|  | Degree of cooperation with Alter |  |  |  |  | -0.10 | 0.55 |
| SCARCITY - PAID WELL (S–PW) | Alter’s cooperation | 0.69 | 0.00 | 0.38 | 0.02 | 0.69 | 0.00 |
|  | Alter’s reputation score |  |  | 0.29 | 0.07 | 0.82 | 0.00 |
|  | Degree of cooperation with Alter |  |  |  |  | 0.26 | 0.10 |

*Note*: ­*significant from Round 10 (corr=0.34, sig=0.03)

**Figure S4 Graphical illustration of correlations between behaviour, gossip, reputation score by treatment**


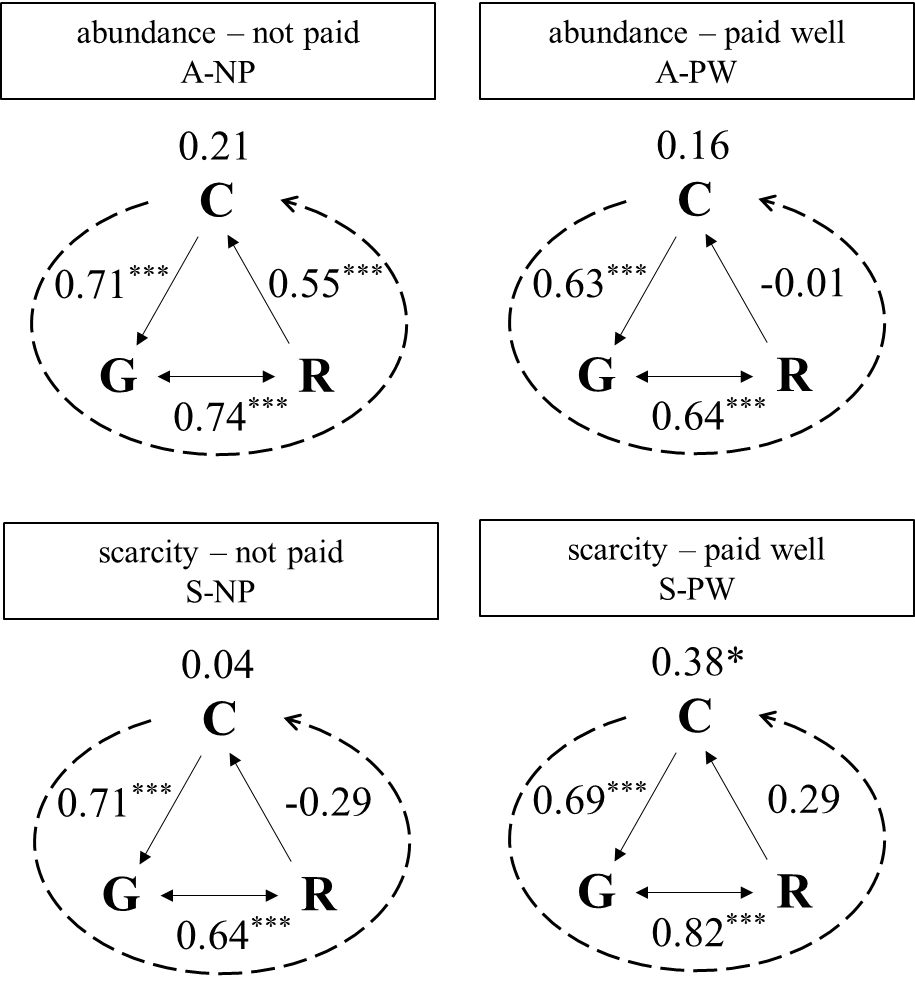


**Table S13 Multilevel mixed-effects logistic regression**

| **Dependent variable: Prisoner's Dilemma choice**  **0 = defect, 1 = cooperate** | **ABUNDANCE - NOT PAID**  **(A–NP)**  **Model 1** | **ABUNDANCE - PAID WELL**  **(A–PW)**  **Model 1** | **SCARCITY - NOT PAID**  **(S–NP)**  **Model 1** | **SCARCITY - PAID WELL**  **(S–PW)**  **Model 1** | **Joint model** |
| --- | --- | --- | --- | --- | --- |
| Alter’s previous cooperative behaviour | 1.1662*** | 0.3444 | 0.352 | 0.9393** |  |
|  | [0.3392] | [0.2999] | [0.3512] | [0.3426] |  |
| Alter’s reputation score |  |  |  |  | 0.0382*** |
|  |  |  |  |  | [0.0041] |
| baseline: ABUNDANCE - NOT PAID (A–NP) |  |  |  |  |  |
| ABUNDANCE - PAID WELL (A–PW) |  |  |  |  | 3.6389** |
|  |  |  |  |  | [1.1171] |
| SCARCITY - NOT PAID (S–NP) |  |  |  |  | -0.0599 |
|  |  |  |  |  | [0.9809] |
| SCARCITY - PAID WELL (S–PW) |  |  |  |  | 1.5402 |
|  |  |  |  |  | [1.1582] |
| Alter’s Reputation score – treatment interaction |  |  |  |  |  |
| ABUNDANCE - PAID WELL (A–PW) * Alter’s Reputation score |  |  |  |  | -0.0278*** |
|  |  |  |  |  | [0.0051] |
| SCARCITY - NOT PAID (S–NP) * Alter’s Reputation score |  |  |  |  | -0.0088 |
|  |  |  |  |  | [0.0060] |
| SCARCITY - PAID WELL (S–PW) * Alter’s Reputation score |  |  |  |  | 0.0021 |
|  |  |  |  |  | [0.0061] |
| Ego’s Reputation score |  |  |  |  | -0.0155 |
|  |  |  |  |  | [0.0141] |
| Ego’s Reputation score – treatment interaction |  |  |  |  |  |
| ABUNDANCE - PAID WELL (A–PW) * Ego’s Reputation score |  |  |  |  | -0.0223 |
|  |  |  |  |  | [0.0184] |
| SCARCITY - NOT PAID (S–NP) * Ego’s Reputation score |  |  |  |  | 0.0178 |
|  |  |  |  |  | [0.0199] |
| SCARCITY - PAID WELL (S–PW) * Ego’s Reputation score |  |  |  |  | -0.02 |
|  |  |  |  |  | [0.0237] |
| Round |  |  |  |  | -0.0314*** |
|  |  |  |  |  | [0.0094] |
| Ego’s cooperation level in Round 1-5 |  |  |  |  | 4.6916*** |
|  |  |  |  |  | [0.6140] |
| _cons | -2.6362*** | -1.5419*** | -2.8155*** | -1.9954*** | -4.4143*** |
|  | [0.3855] | [0.3179] | [0.4941] | [0.3176] | [0.7913] |
| lns1_1_1 | 0.7011*** | 0.5803*** | 0.9446*** | 0.5401*** | 0.6297*** |
|  | [0.1716] | [0.1508] | [0.1729] | [0.1576] | [0.0822] |
| Nr. of groups | 40 | 40 | 40 | 40 | 120 |
| N | 1274 | 1331 | 1257 | 1228 | 5090 |

*Note*: * p<.05; ** p<.01; *** p<.001, standard errors are in brackets, lns1_1_1: random intercept variance between subjects

# A3. Detailed discussion of treatment effects

In the setting with scarce reputational resources that could directly be translated to monetary gains (S-WP), we expected an intense competition between participants by either increasing their own cooperative behaviour or by wrecking the position of others. Therefore, we expected that cooperation will be higher, and competitors will use the opportunity to worsen the position of others by dishonest gossip. Although only in the short term, it seems that in this treatment, participants took this competition more seriously and strived more for achieving their own reputations by cooperation. In addition, we observed less positive gossiping about individuals with high reputation. The dissemination of false information not just contributes to the deterioration of reputation, but it hinders the reliability of the reputation system. Since individuals are only willing to take the risk of cooperative behaviour if the reputation system is reliable, this can have a negative effect on cooperation. Although strategic cooperation disappeared in the long run, the collapse of the reputation system did not happen in the SCARCITY – PAID WELL S-PW treatment. Reputations have preserved their credibility over time despite the possibility of misinformation maybe because positive gossip was more credible and have been taken into greater account in scoring.

The reputation system was unable to increase cooperative behaviour where limited reputational resources were available, but there was no external motivation for reputation (SCARCITY - NOT PAID, S–NP). Since punishment was more lenient and symbolic in a way that it has not been accompanied by payoff reduction, competition may have not been taken seriously by participants. We also found a slightly different pattern in participants’ reliance on gossiping as negative gossip resulted in less score reduction than in other treatments. This may follow from the fact that improvement of relative position in this treatment can be reached not just by cooperation but by sending negative gossip about others, therefore individuals did not entirely believe them.

The use of external incentives without competition (ABUNDANCE - PAID WELL, A–PW) could have hampered the development of a trusted reputation system in various ways. Given unlimited reputational resources, individuals tried to encourage cooperation by sending positive messages and giving high reputation points for everyone, making it impossible to use the reputational system to differentiate cooperative intentions of others. Although participants tended to balance this positivity by reducing reputation scores in a greater extent of those who were a target of a neutral or a negative gossip, they were less dependent on reputation scores during their PD decisions.

Although we do not observe strategic reputation building, the reputational system could supervise decisions where reputation building was not incentivized externally, and the achievement of good reputation was available for everyone (ABUNDANCE - NOT PAID, A–NP), as we found an association between players’ decision in the PD game and partners’ previous behaviour.

# A4. Instructions of the experiment

Welcome to the decision-making experiments organized by the Corvinus University of Budapest!

The decision-making experiments carried out by the Hungarian Academy of Sciences, Centre for Social Sciences, "Lendület" Research Center for Educational and Network Studies (RECENS), led by Károly Takács, and supported by the European Research Council (ERC CoG 648693).

Please turn off your phone or completely turn it down!

In the following, instructions will appear on your screen about the experiment. You participate in the experiment together with people in this room. Instructions are the same for all participants. You get the most important instructions on paper as well. You can use them at any time during the experiment. Please do not take these instructions with you after the experiment, leave them on the table.

VERY IMPORTANT rule is that it is STRICTLY FORBIDDEN to talk to or to signal to others! Violation of this rule may result in disqualification from this experiment.

The experiment takes about 75 minutes. Your payoffs in the experiment will be paid at the end of the experiment.

The amount of your payoff depends on your own choices and the decisions of others. The precise calculation of your payment will be described later in more detail.

By pressing the \ "Next \" button, you agree that your answers will be exclusively and anonymously used for scientific research purposes.

Thank you in advance for your participation!

If you're ready, press the \ "Next \" button! Have fun and good luck!

**INSTRUCTIONS**

The experiment will consist of several rounds of decision-making. In each round, you will be paired with two other participants. Participants of the experiment will be identified with numbers ranging from 1 to 20.

It is important that you do not play with the same participants in each round! Both of your pair determines by a random number generator. The ID of your current pair will be displayed on your screen. The IDs will be kept confidential, neither during the experiment nor at the end of it will we not reveal which identifier participants belonged to.

Each round is important for your final payoff!

1 round in the first phase and 5 rounds in the second phase will be selected using a random number generator. The average winnings in these rounds will be your final payoff. We add everyone 1000 forints as a bonus. Payoffs will be rounded up to HUF 100.

Each pair faces with the following decision-making options. Two options are provided: the two options are indicated with L and R. The amount that you win this round does not depend only on your decision, but also on your partner's decision. It is detailed in the following what payoff can be expected:

If both of you choose L: HUF 1500

If you choose L and your partner select R: HUF 2500

If you choose R and your partner select L: HUF 0

If both of you choose R: HUF 500

If you run out of time: HUF 0

It is important that all the information that you receive is real. The time available to you will be projected in the upper right corner of your screen.

When you are ready, please click on the "Next" button.

**FURTHER INSTRUCTIONS**

The same decision will be taken in the next rounds and the amounts that you can win are the same as previously.

In each round, you will randomly be paired with two other participants. It is therefore important that you do not play with the same participants in each round, but your pair is determined by a random number generator!

The change is that now the participants can be scored from 0 to 100 by you on the basis how reliable they are according to you A maximum of 950 points can be distributed. By default, the starting score is set to 50, as a neutral medium. (So long as you distribute more than the maximum points, you get an error message. If this will not be corrected in time, the total score will be rounded down proportionately.)

The points you receive will be taking into account in the final payoff!

The payoff for that run will be adjusted by your average point.

If the point you received on average corresponding to the neutral value of 50, then your payoff will be unchanged in the current round. In comparison, a one-unit decrease/increase in your average point reduces/increases your payments by HUF 20. For instance, if all other participants give you 0 point, then your payment decreases by HUF 1000. If all other participants give you 100 point, your payment increases by HUF 1000.

In addition to the scoring it is also a new element that one pair from the previous rounds will be randomly selected, and you get acquainted with the decisions they have made in that round.

Then, you'll be able to send a message to another randomly selected participant. The ID of the participant – to whom you can send the message – will appear on your screen. Then, optionally, you can select four participants, the four whom the message is about. There is no cost of sending a message.

The time available for your choice will display in the top right corner of the screen. Attention please! If you run out of time, it is considered that you did not want to send a message!

After all messages have been sent, messages you received will appear on your screen.

Now you can also modify the scores on the 100 points scale that you have given to others indicating how reliable they are.

When you are ready, please click on the "Next" button. Have fun and good luck!

**A5. Screens of one round in the first phase (Round 1-5) of the experiment (with English translation)**


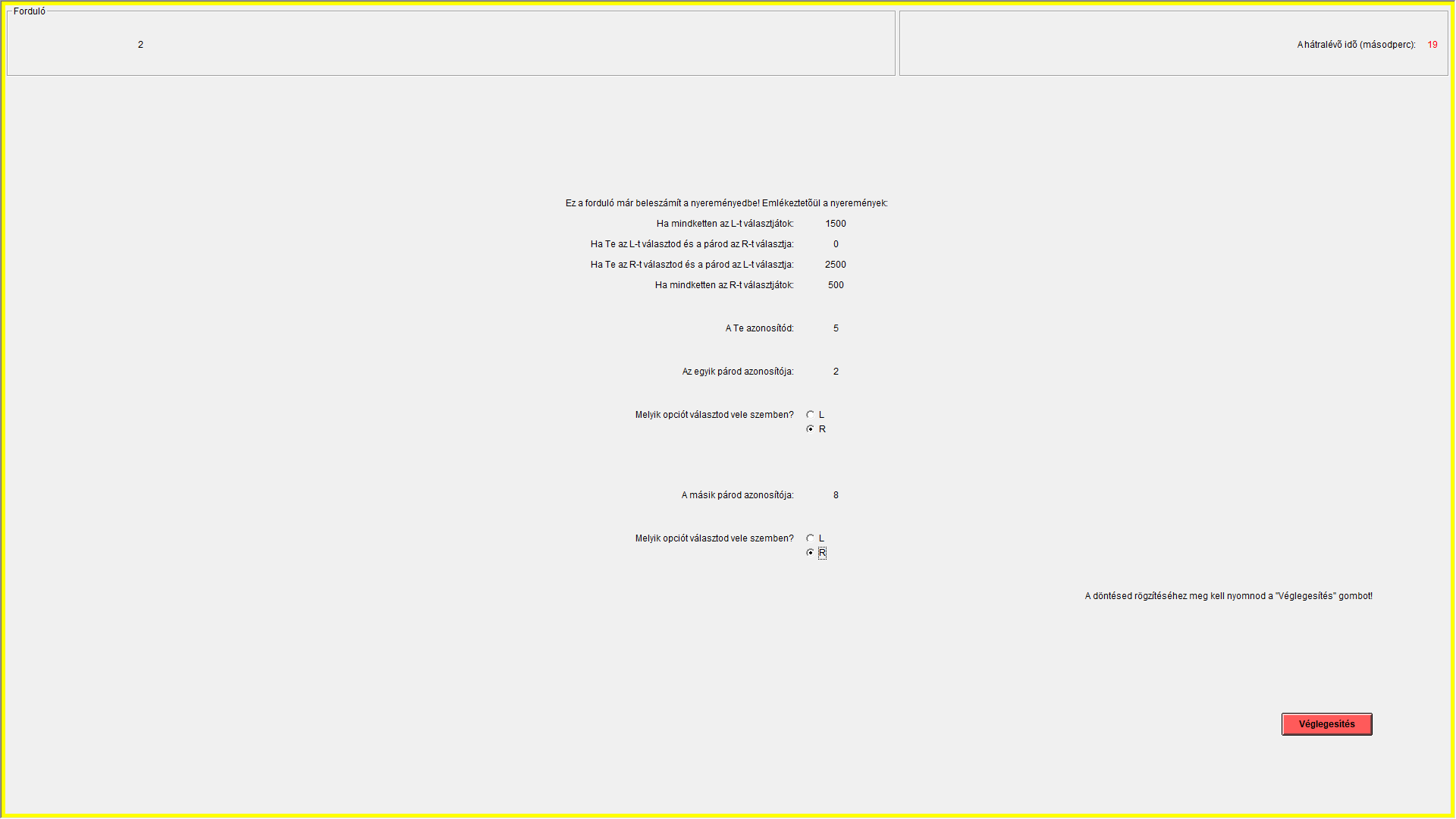


| Round 2 | Remaining time (sec): 19 |
| --- | --- |
| This round may count towards your final payment. Remember, your payoff is:  If both of you choose L: 1500  If you choose L and your partner chooses R: 0  If you choose R and your partner chooses L: 2500  If both of you choose R: 500  Your ID: 5  Your first Partner's ID: 2  Which option do you choose against her/him? L/R  Your second Partner's ID: 8  Which option do you choose against her/him? L/R  To confirm your decision, you need to click on the 'confirm' button.  Confirm | |


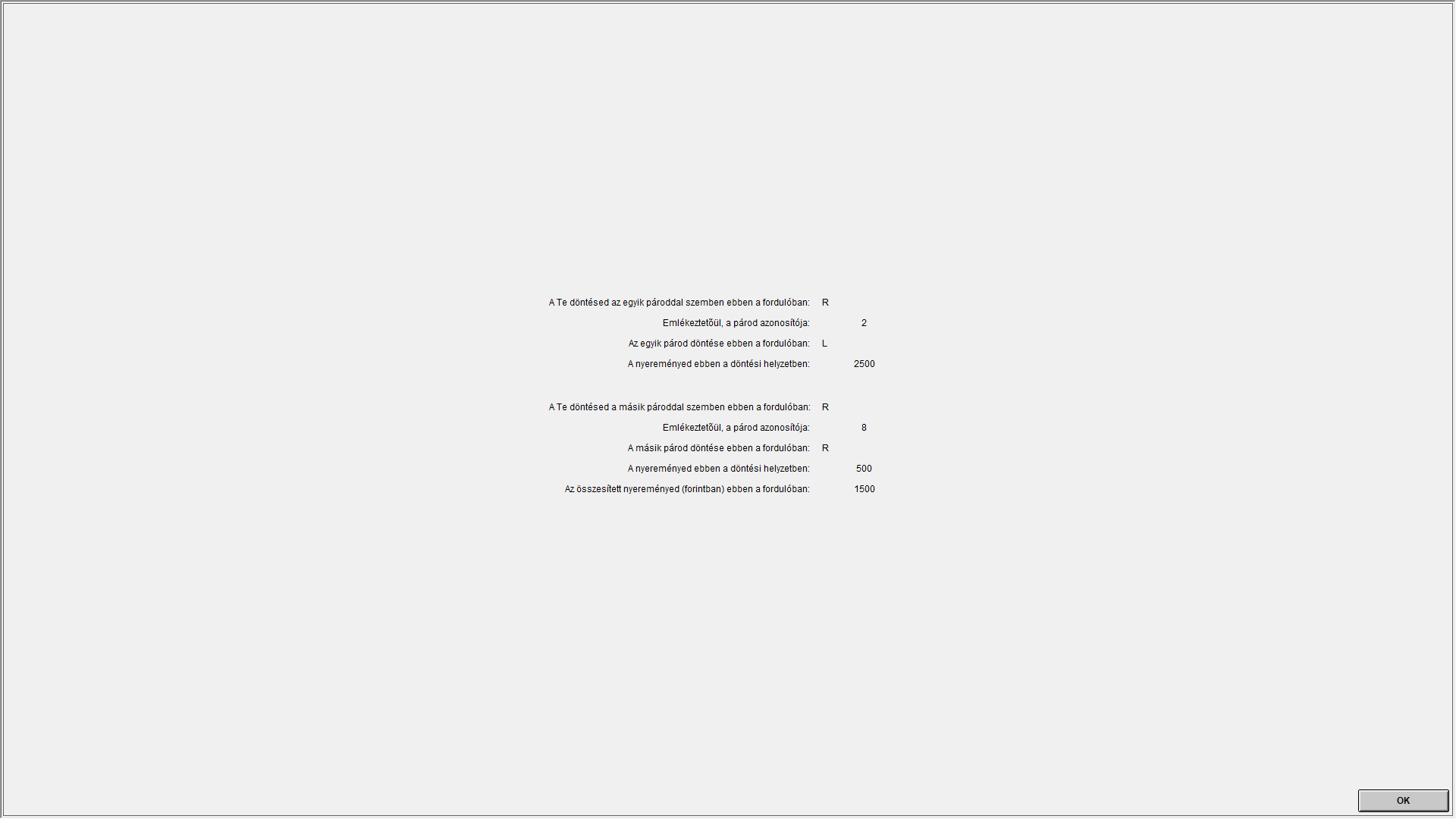


| Your decision against your first partner in this round: R  As a reminder, your partner's ID: 2  Your partner's decision in this round: L  Your payoff in this round: 2500  Your decision against your second partner in this round: R  As a reminder, your partner's ID: 8  Your partner's decision in this round: R  Your payoff in this round: 500  Your combined payoff in this round (in HUF): 1500  OK |
| --- |

**A6. Screens of one round in the second phase (Round 6-22) of the experiment (with English translation)**
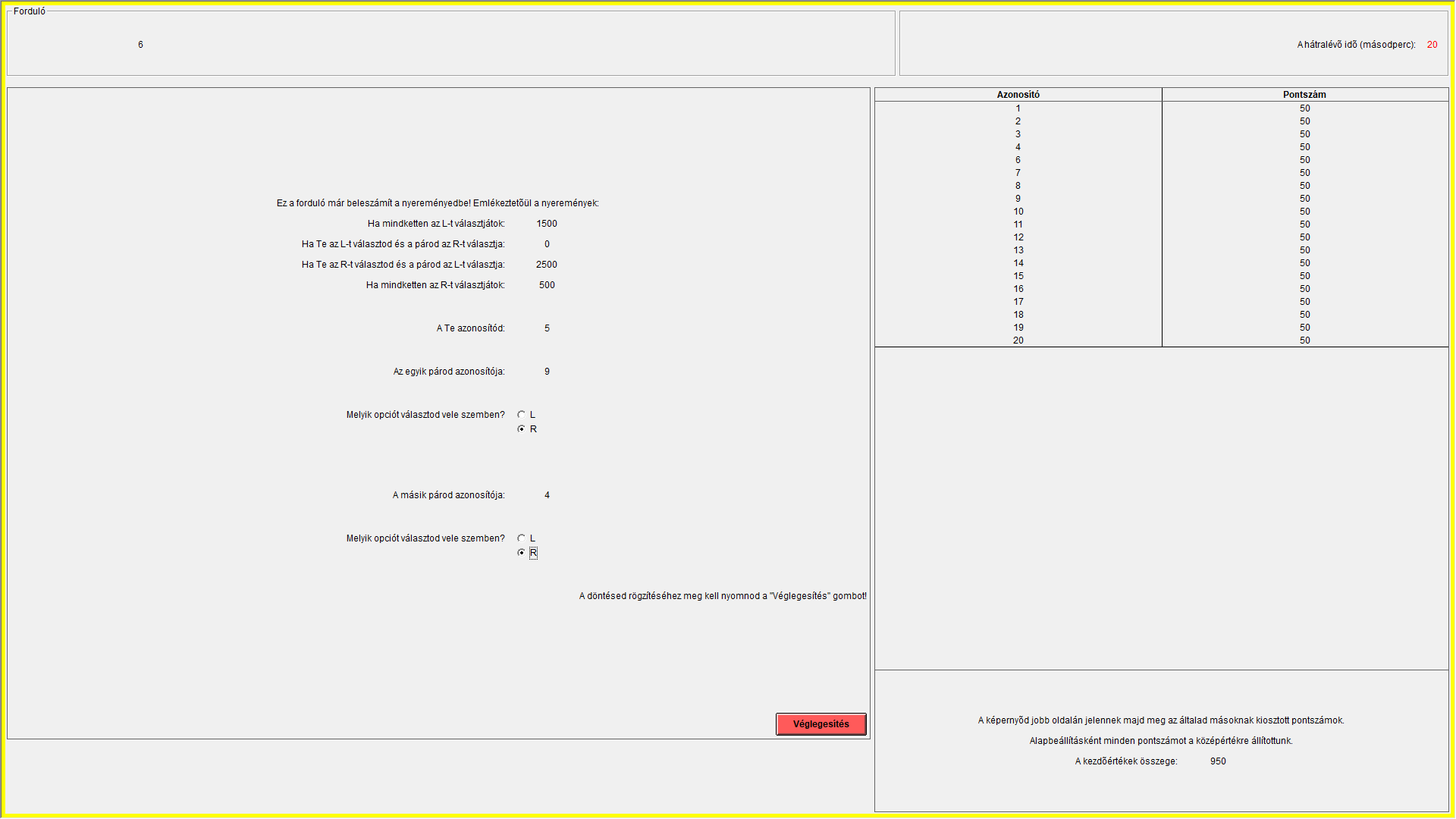


| Round 6 | Remaining time (sec): 20 | |
| --- | --- | --- |
| This round may count towards your final payment. Remember, your payoff is:  If both of you choose L: 1500  If you choose L and your partner chooses R: 0  If you choose R and your partner chooses L: 2500  If both of you choose R: 500  Your ID: 5  Your first Partner's ID: 9  Which option do you choose against her/him? L/R  Your second Partner's ID: 4  Which option do you choose against her/him? L/R  To confirm your decision, you need to click on the 'confirm' button.  Confirm | ID | Scores |
|  | 1  2  3  4  6  7  8  9  10  11  12  13  14  15  16  17  18  19  20 | 50  50  50  50  50  50  50  50  50  50  50  50  50  50  50  50  50  50  50 |
|  |  |  |
|  | The scores you give to others will appear on the right side of your screen.  At the beginning, all scores were set to mean.  The total default value is 950.* | |

*Note:* *From Round 7 the text has changed: ‘The total score allocated by you is: … ’ which text was missing in the ‘abundance treatment’


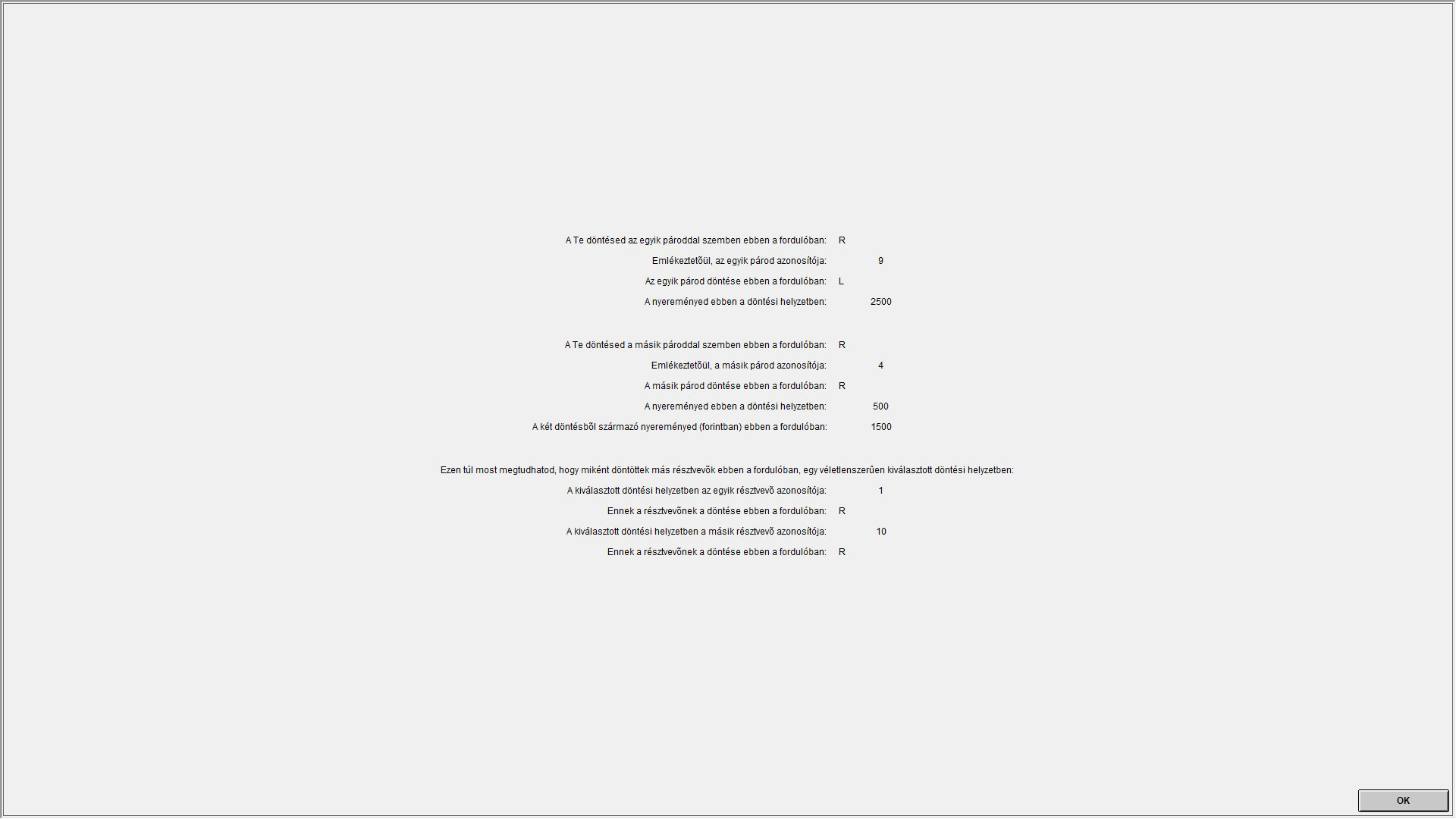


| Your decision against your first partner in this round: R  As a reminder, your partner's ID: 9  Your partner's decision in this round: L  Your payoff in this round: 2500  Your decision against your second partner in this round: R  As a reminder, your partner's ID: 4  Your partner's decision in this round: R  Your payoff in this round: 500  Your combined payoff in this round (in HUF): 1500  Moreover, now you can learn how other participants have decided in this round, in a randomly selected play:  The ID of one participant in the selected play: 1  The decision of this participant in this round: R  The ID of the other participant in the selected play: 10  The decision of this participant in this round: R  OK |
| --- |


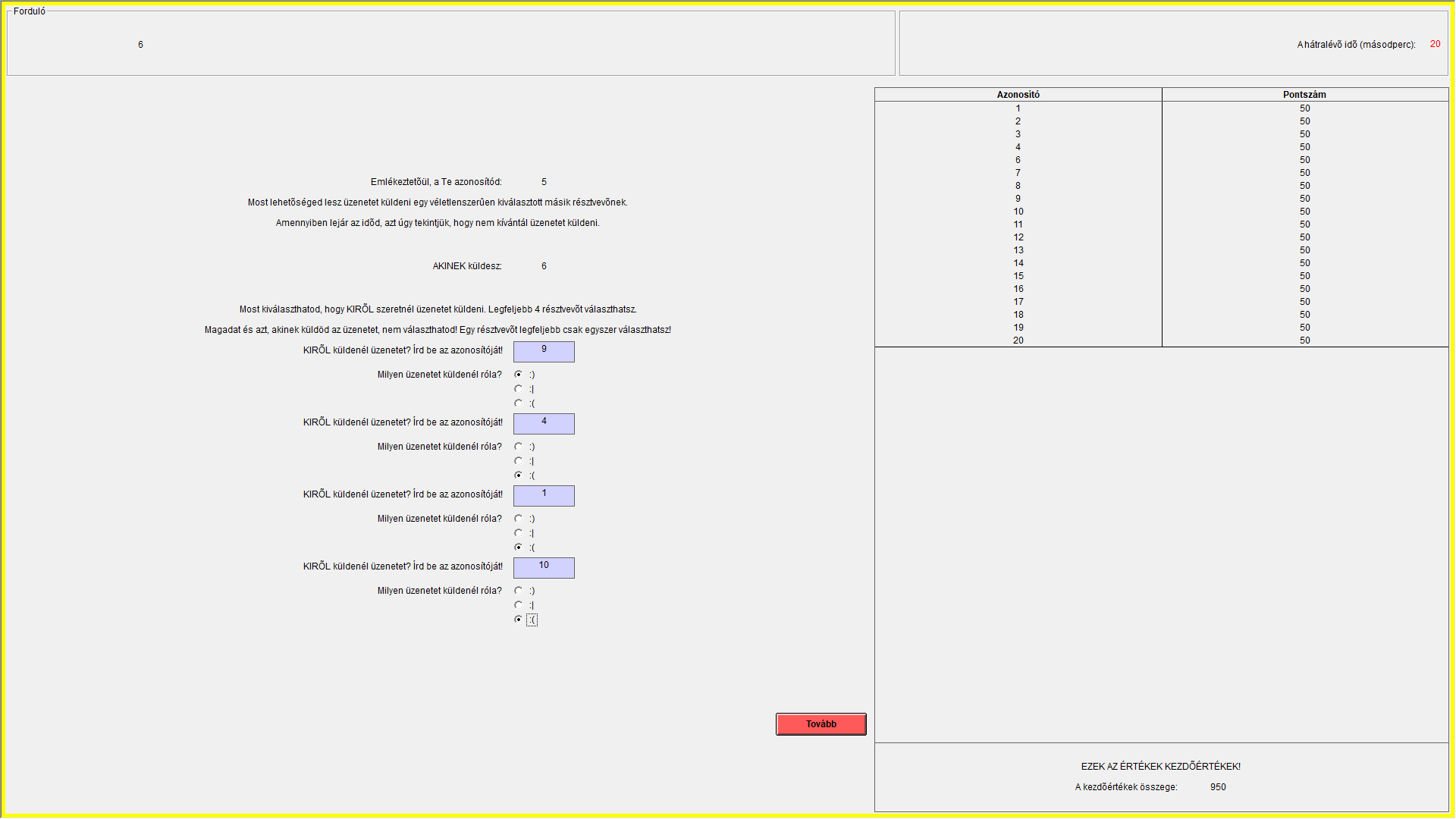


| Round 6 | Remaining time (sec): 20 | |
| --- | --- | --- |
| As a reminder, your ID is: 5  Now you can send messages to a randomly selected participant.  If you run out of time, it is considered that you did not want to send a message.  TO WHOM you send messages: 6  Now you can choose ABOUT WHO you want to send a message. You can select a max. of 4 participants.  You cannot choose yourself and who you are sending the message to. You can select one participant only once.  ABOUT WHO do you want to send a message? Write his/her ID here:  What message would you like to send about him/her? :) / :| / :(  ABOUT WHO do you want to send a message? Write his/her ID here:  What message would you like to send about him/her? :) / :| / :(  ABOUT WHO do you want to send a message? Write his/her ID here:  What message would you like to send about him/her? :) / :| / :(  ABOUT WHO do you want to send a message? Write his/her ID here:  What message would you like to send about him/her? :) / :| / :(  Next | ID | Scores |
|  | 1  2  3  4  6  7  8  9  10  11  12  13  14  15  16  17  18  19  20 | 50  50  50  50  50  50  50  50  50  50  50  50  50  50  50  50  50  50  50 |
|  |  |  |
|  | THESE SCORES ARE DEFAULT VALUES.  The total default value is 950.* | |

*Note:* *From Round 7 the text has changed: ‘The total score allocated by you is: … The maximum scores you can allocate is 950.’ The second sentence appeared only where scarcity was introduced.


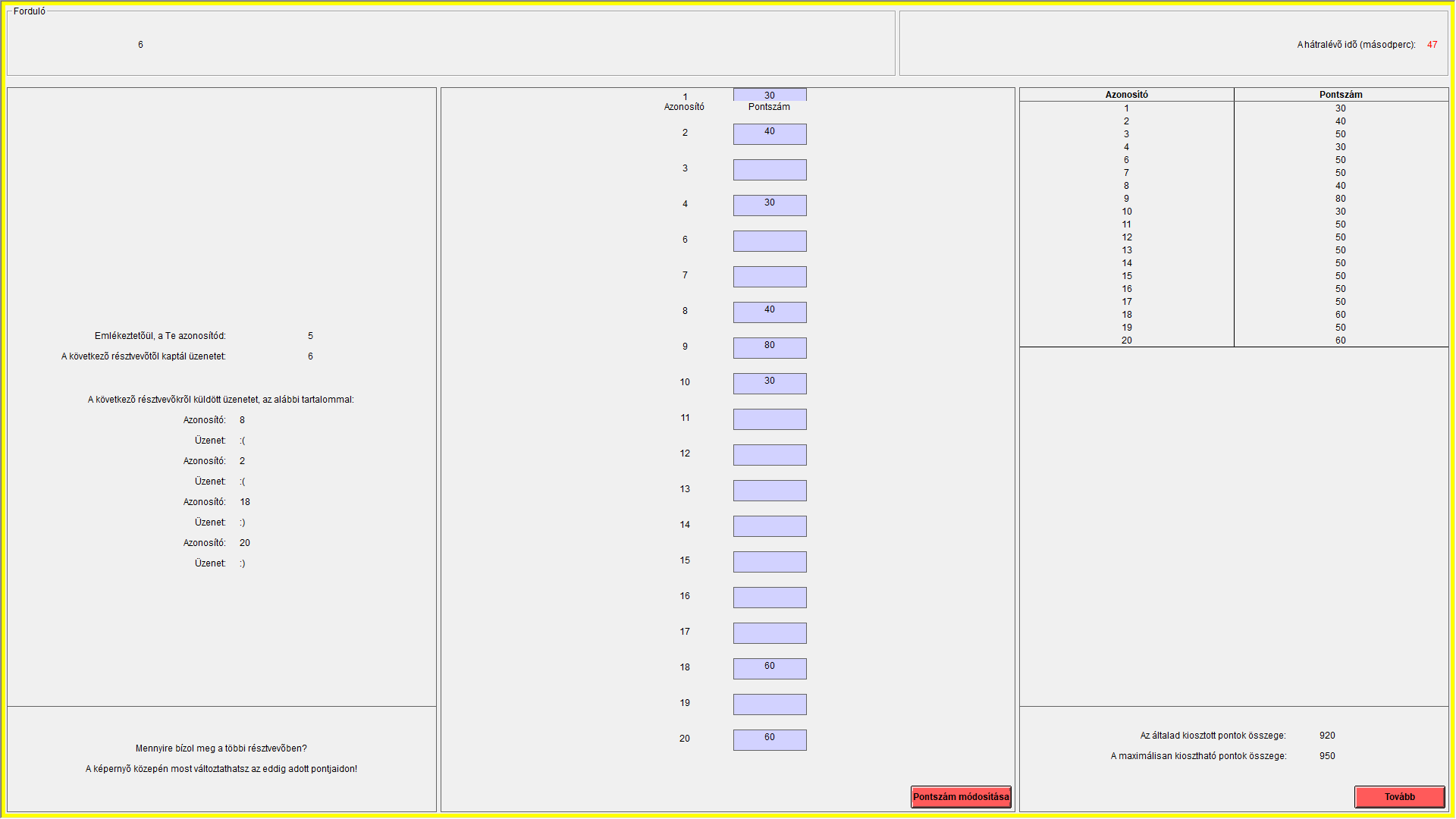


| Round 6 |  | Remaining time (sec): 20 | |
| --- | --- | --- | --- |
| As a reminder, your ID is: 5  You received messages FROM: 6  You received the following messages:  ID: 8  Message: :(  ID: 2  Message: :(  ID: 18  Message: :)  ID: 20  Message: :) | ID Scores  1 30  2 40  3 _  4 30  6 _  7 _  8 40  9 80  10 _30  11 _  12 _  13 _  14 _  15 _  16 _  17 _  18 60  19 _  20 60 | ID | Scores |
|  |  | 1  2  3  4  6  7  8  9  10  11  12  13  14  15  16  17  18  19  20 | 30  40  50  30  50  50  40  80  30  50  50  50  50  50  50  50  60  50  60 |
|  |  |  |  |
| How much do you trust other participants?  Now, in the middle of the screen you can change the scores of other participants. |  | THESE SCORES ARE DEFAULT VALUES.  The total default value is 950. | |

*Note:* *From Round 7 the text has changed: ‘The total score allocated by you is: … The maximum scores you can allocate is 950. ’ The second sentence appeared only where scarcity was introduced.


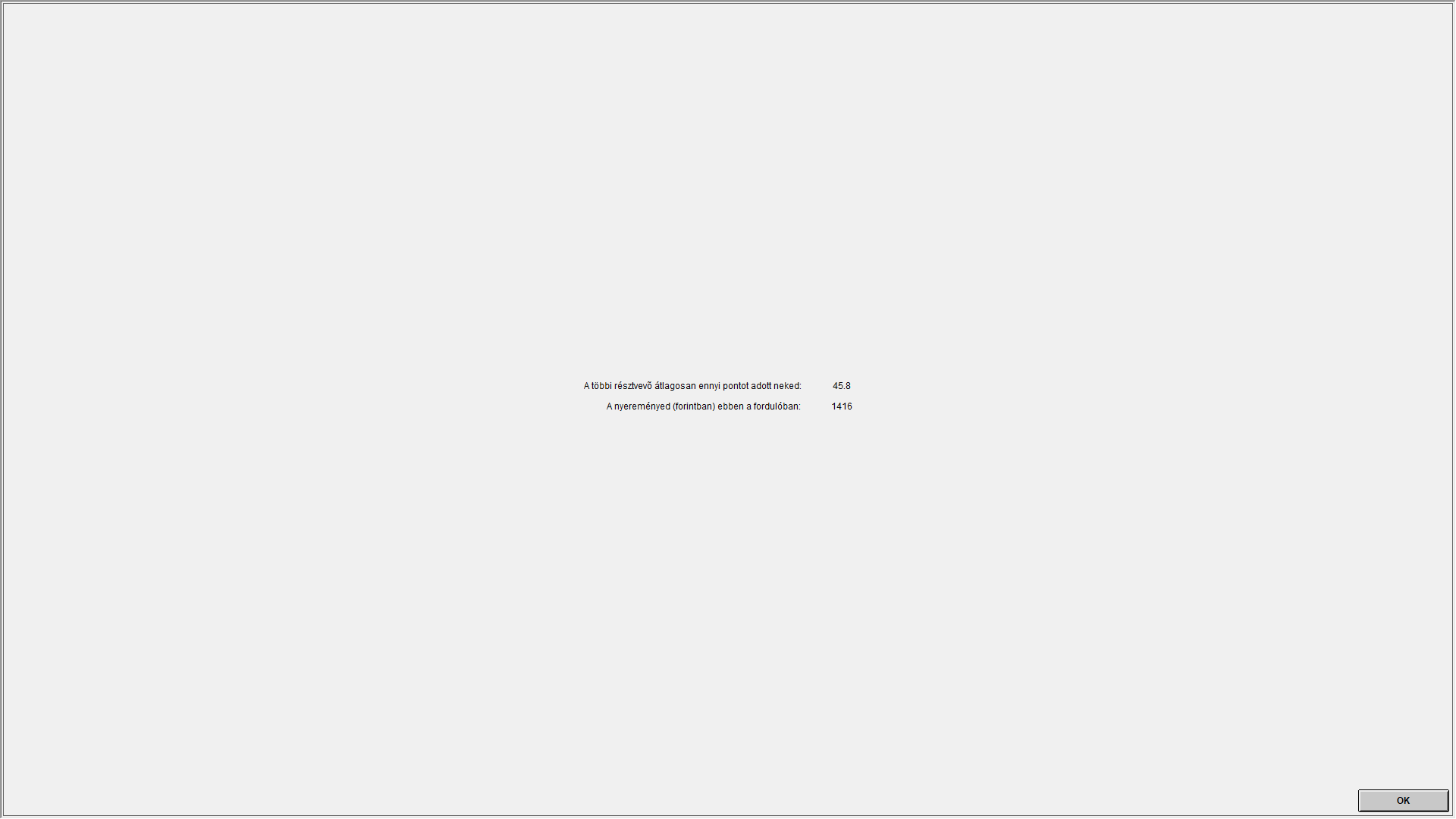


| The score other participants gave you on average: 45.8  Your payoff in this round (in HUF): 1416 |
| --- |

*Note:* *The payoff was reduced/increased only in paid well (PW) treatments.
